# Opto-electronic characterization of third-generation solar cells

**DOI:** 10.1080/14686996.2018.1442091

**Published:** 2018-03-19

**Authors:** Martin Neukom, Simon Züfle, Sandra Jenatsch, Beat Ruhstaller

**Affiliations:** a Institute of Computational Physics, Zurich University of Applied Sciences, Winterthur, Switzerland; b Fluxim AG, Winterthur, Switzerland; c Institute of Physics, University of Augsburg, Augsburg, Germany

**Keywords:** CELIV, OCVD, TPV, DLTS, TPC, IMPS, impedance spectroscopy, charge carrier mobility, organic solar cells, perovskite solar cells, 40 Optical, magnetic and electronic device materials, 209 Solar cell / Photovoltaics

## Abstract

We present an overview of opto-electronic characterization techniques for solar cells including light-induced charge extraction by linearly increasing voltage, impedance spectroscopy, transient photovoltage, charge extraction and more. Guidelines for the interpretation of experimental results are derived based on charge drift-diffusion simulations of solar cells with common performance limitations. It is investigated how nonidealities like charge injection barriers, traps and low mobilities among others manifest themselves in each of the studied cell characterization techniques. Moreover, comprehensive parameter extraction for an organic bulk-heterojunction solar cell comprising PCDTBT:PC_70_BM is demonstrated. The simulations reproduce measured results of 9 different experimental techniques. Parameter correlation is minimized due to the combination of various techniques. Thereby a route to comprehensive and accurate parameter extraction is identified.

## Graphical abstract

**Figure:**
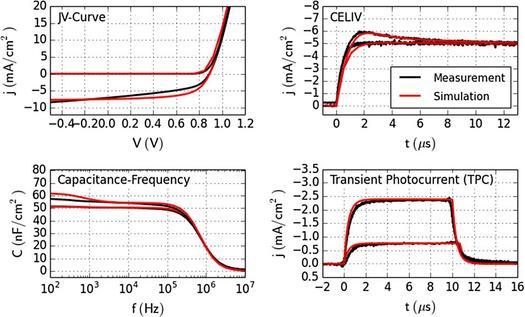

## 1. Introduction

The past decade witnessed an impressive development in power conversion efficiencies of novel thin film solar cells based on organic materials, quantum dots, hybrid and perovskite materials. All these new concepts are denoted by the term ‘third generation photovoltaics’ and have in common that the variety of possible materials and device structures is very large. Accurate characterization is therefore crucial for material screening and device optimization.

Developing a physical understanding of mechanisms governing the operation of third-generation solar cells is much more demanding than for silicon solar cells. Crystalline silicon solar cells are doped and thicker than 100 μm. Both factors combined lead to the screening of the electric field such that the largest part of the device is field-free. Therefore, charge transport is governed by diffusion of minority carriers within the doped region. Consequently, the minority carrier lifetime and diffusion length characterize the quality of crystalline silicon [1–4].

In contrast to crystalline silicon, photogeneration and transport of charges in third-generation solar cells are more difficult to understand and requires more complex characterization techniques. Organic solar cells, for example, are between 50 and 300 nm thick and comprise p-i-n structure (A bulk-heterojunction solar cell can be considered as p-i-n type as the bulk is usually undoped). Electrodes with different workfunctions and doped injection layers create a built-in potential that drops inside the intrinsic region. Charge transport is facilitated by drift in this built-in electric field. Inside the intrinsic region the electron and hole densities vary spatially – there are no clear ‘minority carriers’ like in the bulk of crystalline silicon. Quantifying a diffusion length in a p-i-n structured solar cell is, therefore, not meaningful. Characterizing and quantifying charge transport in p-i-n structures requires measuring the electron and hole mobilities, the recombination coefficient, the built-in potential, charge injection barriers and further parameters associated with charge trapping. It is, however, very difficult to assess these parameters individually, as there are highly entangled in a solar cell device.

Furthermore, material parameters often depend on the processing, the solvents, thermal treatment and the substrate [5]. Material parameters can even depend on the batch [6,7]. For example, metal workfunctions measured by photoelectron spectroscopy might be subject to change when an organic material is deposited on top, due to chemical reactions at the interface [8]. Individual characterization of the ‘ingredients’ of a solar cell is therefore not feasible and comprehensive device characterization is mandatory. There are numerous experimental techniques available to study electrical material and device parameters of solar cells. In this review, we aim to give an overview of some of the most prominent experimental techniques. We use numerical simulation to explain and quantify the effects that are observed in each of these measurements.

To obtain quantitative solar cell and material parameters, the combination of several experimental techniques with numerical simulation is required [9]. The numerical simulation is fitted to the experimental results. In the last chapter of this review, we present measurement and simulation data for an organic solar cell comprising PCDTBT:PC_70_BM as the active layer. We reproduce nine experimental techniques with one set of parameters.

We aim to provide a guide for the interpretation of experimental results. These experiments help to gain qualitative understanding of the underlying physical processes. While in the following we focus on organic solar cells, the characterization techniques discussed here are not restricted to them but can also be applied to other devices as quantum dots or perovskite solar cells.

## 2. Case study

**Figure 1.**
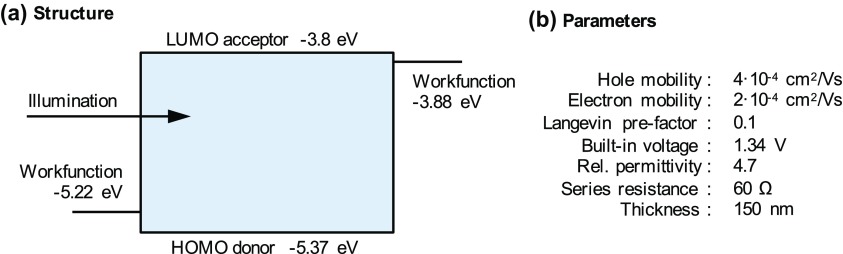
(a) Device structure of the ‘base’ case used in this study. LUMO and HOMO stand for lowest unoccupied and highest occupied molecular orbitals, respectively. (b) Simulation parameters of the ‘base’ case. Full simulation parameters of all cases are listed in the supplemental information (SI).

In order to explain the various effects to be observed in the different experimental techniques we first define 11 cases of solar cells each corresponding to a specific loss mechanism. We first define a ‘base’ case from which all other cases are derived. The ‘base’ case is an organic bulk-heterojunction solar cell as depicted in Figure 1 with a realistic set of parameters similar to the PCDTBT:PC_70_BM device investigated in the last section. All the cases are defined and described in Table 1.

**Table 1.** Definition of 11 cases of solar cells.

Case	Description
Base	This is the standard single-layer device without charge traps or doping. It is 150 nm thick and has Ohmic contacts with low barriers on both sides. All other devices are derived from this base device.
	The detailed set of parameters can be found in the supplemental information (SI).
Extraction barrier	This device features an extraction barrier for electrons. Such a barrier can occur if an oxide layer forms at the electrode. It is modelled by an additional 3 nm thin layer between active material and electron contact with 0.35 eV energy offset. Such oxide formation has for example been shown in P3HT:PCBM solar cells comprising an aluminium electrode [7,10,11].
Non-aligned contact	This device has an injection barrier for electrons of 0.45 eV. This is the case if the workfunction of the metal is too high to match the LUMO level of the active material [12].
Low mobility	The active material has a mobility that is only 10% of the mobility of the base device. Both electron and hole mobilities are reduced, such that the ratio *μ*_*e*_/*μ*_*h*_ remains as in the base device. Low mobilities can for example occur due to a unfavourable donor/acceptor morphology in organic solar cells [5].
High Langevin recombination	The active material has a Langevin recombination efficiency that is 10 times larger than for the base device. The Langevin recombination efficiency depends on the material and on the morphology of bulk-heterojunction solar cells [5]. Phase segregation for example can lead to a lower recombination pre-factor [13].
Shallow traps	The active material has an electron trap density of 3⋅10^17 1^/cm^3^ with a trap-depth of 0.3 eV. In organic solar cells the trap density can depend on material purity [14].
Deep traps	The active material has the same trap density of 3⋅10^17 1^/cm^3^ like ‘shallow traps’ but with a depth of 0.8 eV. This trap is located in the middle of the band-gap and leads to enhanced Shockley-Read-Hall (SRH) recombination [15].
Low shunt resistance	This device has an Ohmic shunt resistance of 50 kΩ (2.25 kΩ⋅cm^2^). Shunt resistances can occur due to non-uniformity of the film, particle contaminations, spikes of the ITO leading to short-circuits, pinholes or others [16]. Shunt resistances can also be non-Ohmic and show SCLC behaviour [17]. For simplicity Ohmic shunting is used here.
High series resistance	The device has an Ohmic series resistance of 350 Ω (15.7 Ω⋅cm^2^). A high series resistance can be caused by the low lateral conductivity of the transparent electrode [18].
High bulk doping density	The bulk of the device is p-doped with 1⋅10^17 1^/cm^3^. Unintentional doping can occur due to impurities that ionize. Very deep traps can have the same effect. Photo-oxidation of single molecules during degradation can also lead to doping [19].
Low charge generation	In this device the photon-to-charge conversion efficiency is reduced to 1/3. The physical origin can be reduced light absorption or hindered exciton dissociation. The latter can be the case if the phase-mixing is too coarse in an organic bulk-heterojunction solar cell [5,20].

Notes: Each case is a set of parameters describing a solar cell with a particular loss mechanism like charge trapping, doping or a shunt resistance. These cases are later used in the simulation of the various experimental techniques. Parameters are described in the SI.

Each case describes a solar cell with a particular performance reduction. The cases are then compared with the base case. These cases correspond to sets of parameters of the drift-diffusion model that are used for the simulation of the various experimental techniques.

Another common performance limitation is an imbalance in electron and hole mobilities. The slower charge carrier type accumulates leading to space-charge and screens the electric field. We show simulations of this additional case in Figure S8 in the supplemental information.

### 2.1. Simulation model

Our model solves the charge drift-diffusion equations on a one-dimensional grid. It incorporates Langevin recombination, trapping and de-trapping, Shockley-Read-Hall (SRH) recombination and doping. Transport levels for electron and holes as well as trap levels are discrete. Charge carrier densities are fixed at the contacts and calculated according to Boltzmann statistics and a single energy level with some offset to the electrode workfunction. The series resistance and the parallel resistance are considered in the simulation. Light absorption is calculated by a transfer matrix method. A list of all parameters and equations can be found in the supplemental information (SI). Our drift-diffusion model is implemented in the simulation software Setfos 4.5 [21]. We have validated this device model with organic solar cells [9,10,22] and perovskite solar cells [23,24] in the past. The same device model is used in the last section of this review to describe several measurements of a PCDTBT:PC_70_BM bulk-heterojunction solar cell to extract relevant electrical device and material parameters.

Please note that our 1D-model is only valid to describe spatially homogeneous devices as it may be assumed for devices with small active area. Devices with large active area and distributed series resistance may be calculated with a 2D plus 1D approach [25].

### 2.2. Current–voltage characteristics of all cases

**Figure 2.**
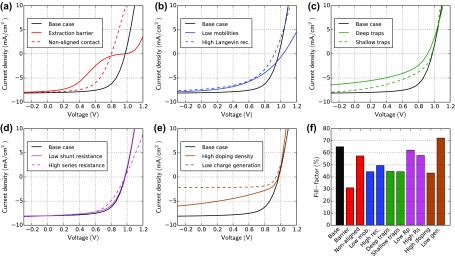
JV-curve simulations for all cases defined in Table 1. (f) The bar-plot shows the fill-factor of all simulated cases. All the described cases impact the fill-factor. It is difficult to identify a specific physical effect if a JV-curve has a low fill-factor.

First of all, we simulate current–voltage (JV) curves under illumination using the cases defined in Table 1. Figure 2 shows the simulation results of all cases in comparison to the base case. In Figure 2(f) the fill-factor of all cases is compared. Bartesaghi et al. showed that the fill-factor in organic solar cells is mainly determined by the ratio of charge extraction versus charge recombination [26].

The case ‘extraction barrier’ shows a pronounced S-shaped JV-curve. S-shapes are often associated with interface effects [7,12,27], which is confirmed here. A non-aligned contact leads to a lower built-in and open-circuit voltage. The JV-curve is therefore shifted to the left.

The open-circuit voltage increases in the case ‘low mobilities’. With Langevin recombination a lower mobility leads to less recombination and thereby to an increase in open-circuit voltage. The charge transport, however, is less efficient leading to a low fill-factor. In the case ‘high Langevin rec.’, the open-circuit voltage and the fill-factor are reduced. A very similar effect occurs in the case ‘deep traps’. The traps are located in the middle of the band-gap leading to efficient SRH-recombination [15]. In contrast to the case ‘high Langevin rec.’ the short-circuit current density is also reduced. ‘Shallow traps’ have no impact on the steady-state short-circuit current in our model, but reduce the fill-factor. Shallow traps lead to a decrease of the effective charge carrier mobility due to capture and release events. If charges are slower their density increases and so does the recombination.

The shunt resistance in this example has only a minor effect on the JV-curve. The fill-factor is slightly reduced since some of the current goes through the parallel resistance instead of the external circuit. A change in series resistance leads to a change in the current slope in forward direction and to a lower fill-factor. A high series resistance is detrimental to device performance since current-flow leads to a voltage-drop over the resistance. The open-circuit voltage is unaffected by the series resistance because the current is zero at this point.

Charge carrier doping can be very detrimental to the efficiency of solar cells as shown by Dibb et al. [28]. Doping introduces extra charge inside the bulk that screens the electric field. This hinders charge extraction and leads to a reduction in photocurrent. This is observed in the case ‘high doping density’. The effect is more prominent for thicker devices [7,28]. In the case ‘low charge generation’ the short-circuit current is decreased as expected and the fill-factor is increased. The forward injection current is unchanged in our example.

We note that several of the investigated cases lead to a similar modification of the JV-curve compared to the base case, as shown in Figure 2. Therefore, by measuring JV-curves only it is hardly possible to identify which non-ideal case is present. In real measurements different effects often occur combined, which renders it even harder to distinguish between them by a single JV-curve. Still, in literature conclusions on charge transport are often drawn by looking at illuminated JV-curves only [22,29–32]. This can be prone to errors. Performing further measurement techniques in the steady-state, transient and frequency domain gives more insight into charge transport physics as will be presented in the next sections.

## 3. Characterization techniques

### 3.1. Dark current–voltage characteristics

Information about the recombination type (so-called ideality) and the shunt resistance can be obtained from current–voltage (JV) curves measured in the dark. In classical semiconductor physics the JV characteristics of a p-n junction in the dark is described by the Shockley equation

(1) $$j\left(V\right)=j_{s}\cdot \left(\exp\left(\frac{q}{n_{\text{idd}}\cdot k_{B}\cdot T}\cdot V\right)-1\right),$$

where *j* is the current density, *j*_*s*_ is the dark saturation current density, *q* is the unit charge, *V* is the voltage, *n*_idd_ is the dark ideality factor, *k*_*B*_ the Boltzmann constant and *T* the temperature. When the transport resistance *R*_*T*_ and the parallel resistance *R*_*p*_ are included the JV-curve is described by the following implicit equation

(2) $$j=j_{s}\cdot \left(\exp\left(\frac{q}{n_{\text{idd}}\cdot k_{B}\cdot T}\cdot \left(V-R_{T}\cdot j\cdot S\right)\right)-1\right)+\frac{V-R_{T}\cdot j\cdot S}{R_{p}},$$

where *S* is the area of the device. Equation (2) needs to be evaluated numerically as no analytical solution can be found. The equation can be fitted to measured dark JV-curves to extract the dark saturation current *j*_*s*_, the dark ideality factor *n*_idd_ and the parallel resistance *R*_*p*_. We clearly distinguish between transport resistance *R*_*T*_ and series resistance *R*_*S*_. The series resistance causing the RC-effects visible in impedance spectroscopy or light-induced charge extraction by linearly increasing voltage (photo-CELIV) measurements is an Ohmic external resistance. It is often caused by the lateral conductivity of the transparent electrode [18]. The transport resistance used in Equation (2) is a resistance that represents the charge transport in the device [33]. The transport resistance is therefore higher than the pure series resistance.

In reverse direction the diode is ideally blocking and the current is determined by the parallel resistance *R*_*p*_. A low parallel resistance is usually caused by shunts in the device [16] and can also be non-Ohmic [17]. Very high trap densities can however also lead to an increase in the reverse current [14]. In forward direction charge carriers are injected and recombine. Charge carriers either recombine in the bulk or travel to the opposite electrode and recombine at the interface. If only one charge carrier type is injected (for example in unipolar devices), the device is either injection limited or space-charge limited [8]. In the latter case the charge carrier mobility can be determined using the Mott-Gurney equation [34]. In solar cells often the dark ideality factor *n*_idd_ is determined from the exponential current-slope in forward direction. It is usually a factor between 1 and 2. In p-i-n solar cells, an ideality factor of 1 is interpreted as bimolecular recombination, a value near 2 is a signature of SRH recombination. The ideality factor is discussed in more detail in the next section.

**Figure 3.**
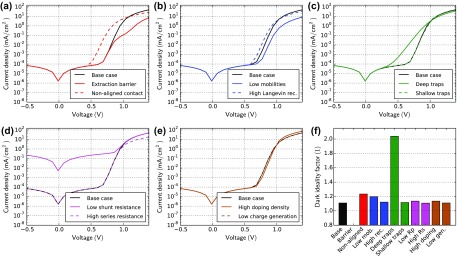
Dark JV-curve simulations for all cases in Table 1. (f) Dark ideality factors are extracted using Equation (2).

Figure 3 shows dark JV-curve simulations of all cases. In these simulations reverse charge injection is negligible. The reverse current is solely determined by the parallel resistance. The case ‘low shunt resistance’ (d) shows much higher reverse current. The parallel resistance can be determined accurately from the differential resistance in reverse.

In the case ‘non-aligned contact’ (a), the exponential current increase is shifted to lower voltage due to the smaller built-in voltage. A low mobility leads to a smaller forward current as observed in Figure 3(b). The slope of the current in the exponential regime is similar in all cases except for the case ‘deep traps’ (c). The dark ideality factor, extracted using Equation (2), is around 1 for most cases and around 2 for the case ‘deep traps’.

It has been shown that the dark ideality factor can be inconsistent with the light ideality factor (next section) and the interpretation can be difficult [35,36]. A high series resistance or low parallel resistance can influence the extraction of the dark-ideality factor [36]. Nevertheless, our simulation results show a clear difference in the dark ideality for the case ‘deep traps’ – the only case with SRH recombination.

### 3.2. Open-circuit voltage versus light intensity

Measuring the open-circuit voltage versus the light intensity can be used to extract the light ideality factor. The ideality factor is a measure whether the recombination type is SRH (*n*_idL_ = 2) or bimolecular (*n*_idL_ = 1). In an ideal device the light ideality factor *n*_idL_ is identical with the dark ideality factor *n*_idd_ from dark JV-curves. In real devices, dark and light ideality factor can deviate. Since the light ideality factor is not influenced by the series resistance it is easier to analyse [36].

An expression for the open-circuit voltage *V*_oc_ is obtained by setting the current to zero in the Shockley equation (Equation (1))

(3) $$V_{\text{oc}}=n_{\text{idL}}\cdot \frac{k_{B}\cdot T}{q}\cdot \ln\left(\frac{j_{\text{ph}}}{j_{s}}+1\right),$$

where *n*_idL_ is the light ideality factor, *k*_*B*_ the Boltzmann constant, *T* the temperature, *q* the unit charge, *j*_ph_ the photocurrent and *j*_*s*_ the dark saturation current. Under the assumption that the photocurrent scales linearly with the light intensity and *j*_ph_/*j*_*s*_ >> 1, we obtain

(4) $$V_{\text{oc}}=n_{\text{idL}}\cdot \frac{k_{B}\cdot T}{q}\cdot \ln\left(L\right)+C_{1}\left(T\right),$$

where *L* is the normalized light intensity and *C*_1_ is a temperature factor that does not depend on *L*. Please note that *C*_1_ is constant with illumination but not constant with the temperature. The open-circuit voltage decreases with temperature and increases with light intensity. The slope of the open-circuit voltage versus light intensity depends only on the light ideality factor and the temperature. The light ideality factor is calculated according to

(5) $$n_{\text{idL}}=\frac{q}{k_{B}\cdot T}\cdot \frac{dV_{\text{oc}}}{d\left(\ln\left(L\right)\right)}.$$

The light ideality factor *n*_idL_ can further depend on the light intensity. SRH recombination for example is more prominent at low light intensities. Often the average is calculated to obtain a single number for the ideality. It is, however, also interesting to study and compare the ideality factor versus the open-circuit voltage [36].

Generally, an ideality factor of 1 is attributed to bimolecular recombination (radiative recombination), whereas an ideality factor of 2 is attributed to dominant SRH recombination [37]. We however want to point out that the concept is based on a single zero-dimensional device model. In a real device the charge carrier distribution varies in space and energy which can influence the ideality factor even if no traps are present. In organic solar cells, the photocurrent *j*_ph_ can depend on the voltage due to Onsager-Braun dissociation of excitons into free carriers [38]. In devices with field-dependent charge generation, the analysis of the light ideality factor might be prone to errors [39].

**Figure 4.**
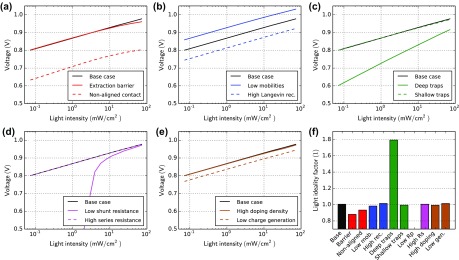
Simulation of the open-circuit voltage dependent on the light intensity for all cases in Table 1. (f) Light ideality factors obtained from the simulation results – an average is used.

Figure 4 shows simulated open-circuit voltages versus light intensity for the different cases. In Figure 4(f), the light ideality factor is shown, calculated from the average slope of the *V*_oc_ versus the light intensity according to Equation (5). The base case has an ideality factor of exactly one. Apart from the case ‘low shunt resistance’ and ‘deep traps’ the ideality factor is around 1. If the recombination pre-factor is increased (b), the *V*_oc_ is lower, but the *V*_oc_-slope remains constant. In the case of ‘deep traps’ (c), the slope (*V*_oc_ vs. *L*) is significantly steeper leading to an average ideality factor of 1.8. In the case ‘low shunt resistance’ (d), the *V*_oc_ collapses at lower light intensity and the calculation of an average ideality factor does not make sense.

Thus, our simulation results show that the light ideality factor can be useful to investigate whether SRH-recombination is significant in a device, as we found for the case ‘deep traps’. The analysis works only if the effect is not concealed by a low shunt resistance.

### 3.3. Charge extraction by linearly increasing voltage

CELIV is a popular technique to estimate charge carrier mobilities in thin-film solar cells. It was introduced by Juška et al. [40] in 2000 and many adaptions or extensions were proposed [41–44].

**Figure 5.**
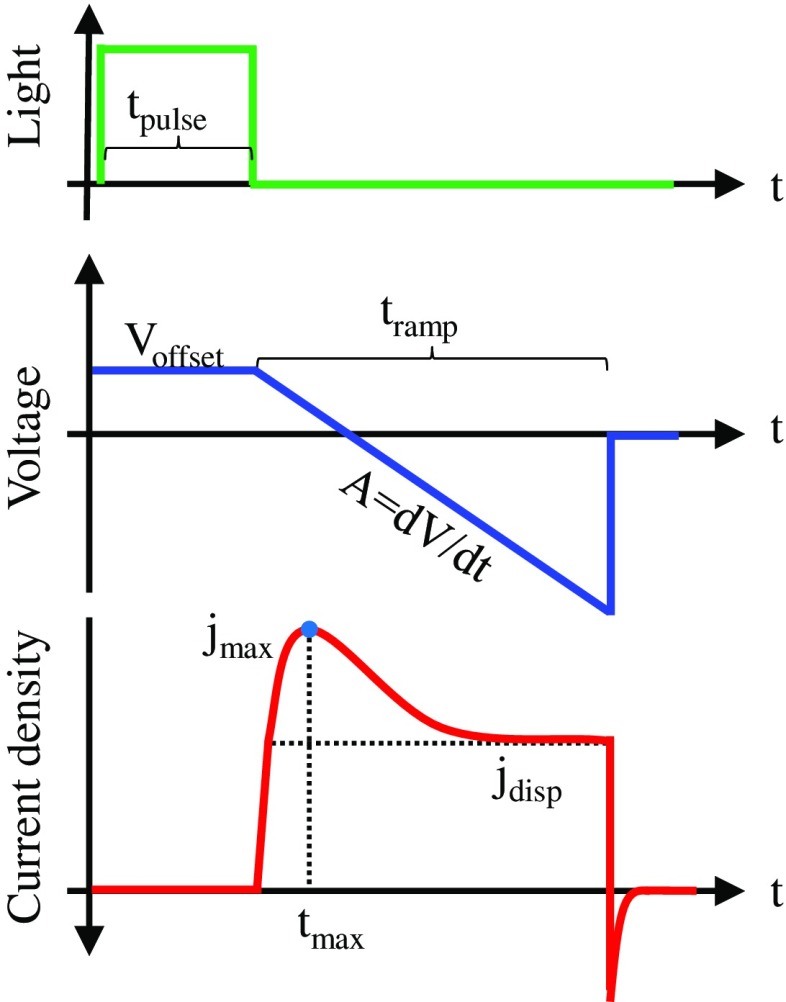
Schematic illustration of a photo-CELIV experiment. The linearly increasing voltage extracts charge carriers and leads to a peak (*j*_max_) in current. The charge carrier mobility is calculated using *t*_max_.

Figure 5 shows the principle of CELIV schematically. A linearly increasing voltage in reverse direction is applied to the device *V*(*t*) = *A*⋅*t*, where *A* is the ramp rate. The linearly changing voltage induces a constant displacement current density *j*_disp_, which is calculated according to

(6) $$j_{\text{disp}}=\frac{1}{S}\cdot \frac{\mathrm{dV}}{\mathrm{dt}}\cdot C_{\text{geom}}=\frac{1}{S}\cdot \frac{d}{\mathrm{dt}}\left(A\cdot t\right)\cdot \frac{S\cdot \varepsilon_{0}\cdot \varepsilon_{r}}{d}=\frac{A\cdot \varepsilon_{0}\cdot \varepsilon_{r}}{d},$$

where *S* is the device area, *C*_geom_ is the geometric capacitance, *ε*_0_ is the vacuum permittivity, *ε*_*r*_ is the relative dielectric permittivity and *d* is the active layer thickness.

If charge carriers are present in the device, they are extracted and lead to a peak in the transient current. According to the time of the current-peak (*t*_max_) the charge carrier mobility can be estimated.

The charges that are extracted by the voltage ramp can be intrinsic (dark-CELIV), be generated by illumination prior to extraction (photo-CELIV) or be injected by a positive voltage prior to extraction (injection-CELIV).

Performing the latter with metal–insulator–semiconductor (MIS) devices allows distinguishing between extracted electrons and extracted holes (MIS-CELIV). Here, the charge dynamics are different and another formula is applied to extract the charge carrier mobility [45–47]. Because the deposition of a thin, high-quality dielectric layer is difficult we demonstrated MIS-CELIV using polar tris(8-hydroxyquinolinato)aluminium (Alq_3_) [48,49].

#### 3.3.1. Dark-CELIV

Dark-CELIV can be used to extract the relative dielectric permittivity and estimate the doping density. The relative dielectric permittivity can be calculated from the displacement current *j*_disp_ by rearranging Equation (6):

(7) $$\varepsilon_{r}=\frac{j_{\text{disp}}\cdot d}{A\cdot \varepsilon_{0}}.$$

The doping density of the device can be estimated by integrating the current. The charges on the electrodes (*Q* = *C*⋅*V*) need to be subtracted. The doping density can be estimated according to

(8) $$n_{\text{doping}}=\frac{1}{d\cdot q}\cdot \left(-\int_{0}^{t_{\text{ramp}}}j\left(t\right)\cdot dt-\frac{C_{\text{geom}}\cdot V\left(t_{\text{ramp}}\right)}{S}\right),$$

where *d* is the active layer thickness, *q* is the unit charge, *t*_ramp_ is the time when the ramp ends, *j* is the current, *C*_geom_ is the geometric capacitance, *V* is the applied voltage and *S* is the device area.

**Figure 6.**
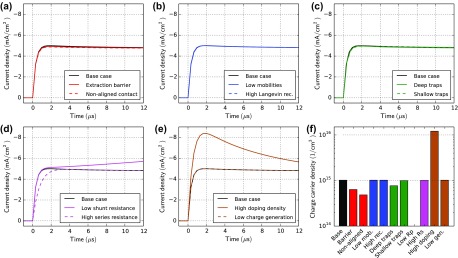
Dark-CELIV simulations of all cases in Table 1. The ramp starts at *t* = 0 with a ramp rate of 171 V/ms. (f) The bar plot shows the extracted charge carrier density.

Figure 6 shows the simulation results of dark-CELIV using the cases defined in Table 1. The only device that shows a current peak is the case with a high doping density. The homogeneous immobile doping induces oppositely charged carriers, which are mobile and can be extracted by CELIV. The parallel resistance leads to an increase in current over time. In such a case neither the integration of the current nor the estimation of the electric permittivity works.

In the other cases mostly RC-effects are observed. We apply Equation (7) to the simulation results. The relative permittivity is obtained with an error of less than 1% in all cases except ‘low shunt resistance’ and ‘high doping density’. Please be aware that the capacitance of the device can change over time, for example due to mobile ions as observed in perovskite solar cells [50–53]. In such a case the calculation of the relative permittivity is less accurate.

The extracted doping density is shown in the bar-plot in Figure 6(f). For the case with high doping, a charge carrier density of 1.2⋅10^16 1^/cm^3^ is extracted. It is almost an order of magnitude lower than the doping density defined as simulation input (1⋅10^17 1^/cm^3^). The reason is that not all charge carriers can be extracted due to the finite ramp-time. The doping density extracted from dark-CELIV should therefore be interpreted as a lower limit for the doping density. We recommend to perform the experiment with varying the ramp-rates and to use the highest density value.

An alternative method to extract the doping density from dark-CELIV currents was presented by Sandberg et al. analysing the shape of the current-decay, based on the Mott–Schottky formalism [54]. Seemann and co-workers demonstrated the evolution of unintentional doping during device degradation using dark-CELIV measurements [19]. In organic solar cells doping is usually detrimental to device performance [28].

#### 3.3.2. Photo-CELIV

In photo-CELIV, free charge carriers are generated by a light pulse and are subsequently extracted by a voltage ramp. As a light source either a light emitting diode (LED) or a laser is used. When the charge carriers are extracted from the bulk they create a current overshoot Δ*j* = *j*_max_−*j*_0_. According to Juška et al. [40], the time where the current peaks (*t*_max_) can be used to calculate the charge carrier mobility by

(9) $$\mu =\frac{2\cdot d^{2}}{3\cdot A\cdot t_{\text{max}}^{2}}\cdot \frac{1}{1+0.36\cdot \frac{\Delta j}{j_{\text{disp}}}},$$

where *μ* is the charge carrier mobility, *d* is the active layer thickness, *A* is the ramp rate, *t*_max_ is the time where the current peaks, *j*_disp_ is the displacement current and Δ*j* is the peak current minus the displacement current. The factor 1 + 0.36⋅Δ*j*/*j*_disp_ in the formula is an empirical correction accounting for the redistribution of the electric field. Bange et al. presented a new equation for the CELIV mobility evaluation validated using drift-diffusion calculations [42]. Lorrmann et al. presented a parametric equation that needs to be evaluated computationally [43]. These adaptions did, however, not lead to an overall improvement of the accuracy of the estimated mobility when applied to our simulation results.

The analytical approach is based on a simple model that considers one charge carrier type to be mobile and the other one to be static. The initial distribution of the charges is considered to be uniform in the bulk and diffusion is neglected. As these approximations are usually inadequate to describe thin film devices, it is apparent that the charge carrier mobility determined based on this model is less accurate compared to full drift-diffusion parameter extraction. In a previous publication, we have studied the CELIV experiment in detail and concluded that the formula (Equation (9)) obtains the charge carrier mobility with an accuracy of a factor of 4. The RC-effects lead to a strong underestimation of the mobility [55]. In such a case, it is advised to increase the thickness of the transparent conducting oxide (TCO) and metallize the TCO stripes. This effectively reduces the series resistance and thereby the RC time constant. Furthermore, it is advised to use devices with a small area leading to a small capacitance and a low RC time.

**Figure 7.**
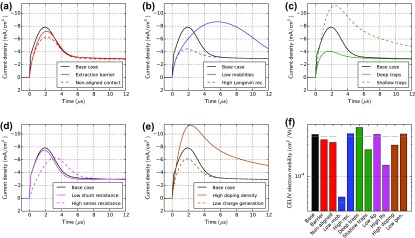
Photo-CELIV simulations for all cases in Table 1. The light is turned off at *t* = 0 and the voltage ramp starts at *t* = 0 with a ramp rate of 100 V/ms. The voltage offset prior the ramp is set such that the current is zero at *t* < 0. (f) The bar plot shows the charge carrier mobility calculated from the peak position (*t*_max_) using Equation (9). The grey lines indicate the electron mobility used as simulation input.

Figure 7 shows photo-CELIV simulation results of all cases defined in Table 1. All devices show a current overshoot with peak-times ranging between 2 and 6 μs. Figure 7(f) shows mobilities calculated using Equation (9). The extracted mobility agrees within a factor of 2 with the input electron mobility (grey line), except for the case with the high series resistance. It leads to a slower charge extraction and to an underestimation of the mobility. In the case of low mobility (Figure 7(b)) the current extraction is slower and the extracted mobility is lower. Traps significantly influence the charge extraction as visible in Figure 7(c). Deep traps create additional recombination channels (SRH), therefore less charge is extracted. Shallow traps, however, save charges from recombination. Therefore, more charge is extracted and the apparent mobility is lower. A similar effect of a slower charge extraction is observed in case of imbalanced mobilities as shown in Figure S8 in the SI.

Photo-CELIV can also be used to estimate the recombination coefficient. Hereby, the experiment is performed several times with varied delay-time between the light pulse turn-off and the voltage ramp start. Then the extracted charge carrier density is plotted versus the delay-time. The recombination coefficient is obtained by fitting a simple zero-dimensional rate equation (d*n*/d*t* = −*k*_2_⋅*n* ^2^ − *k*_1_⋅*n*) [41,56].

If the applied voltage is constant during the delay-time, charge is either injected (if the voltage is too high) or charge is extracted (if the voltage is too low). To keep the cell at open-circuit during the delay-time Clarke et al. used a very fast electrical switch [57]. An alternative that might be easier to realize was proposed by Baumann and co-workers and named OTRACE [44]. Thereby the photovoltage decay is measured first. This voltage signal is then applied during the delay-time of the CELIV experiment. OTRACE ensures that charge carriers remain and recombine in the device during the delay-time and therefore increases the accuracy of the experiment [44].

### 3.4. Transient photovoltage and open-circuit voltage decay

Under open-circuit condition the external current is zero and hence charge generation is equal to charge recombination. Techniques probing the device under open-circuit are generally suited to study recombination. Open-circuit voltage decay (OCVD, sometimes also called large-signal TPV) measurements reveal information about recombination and the shunt resistance. In OCVD measurements, the device is first illuminated by an LED or a laser to create charge carriers. Then the light is turned-off and the decay of the voltage is measured over time.

**Figure 8.**
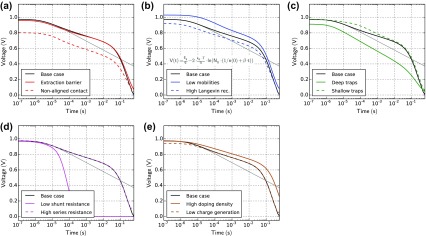
OCVD simulations for all cases in Table 1. The light is turned off at *t* = 0. The grey line indicates the analytic solution (Equation (11)) assuming homogeneous charge densities and purely bimolecular recombination.

Figure 8 shows OCVD simulation results of the defined cases. All the cases have in common, that the voltage drops significantly beyond 50 ms after light turn-off. This is related to the shunt resistance. The most pronounced effect with respect to the ‘base’ case is visible in the case ‘low shunt resistance’ (Figure 8(d)). Instead of recombining slowly the charges flow through the shunt resistance and deplete the device. When the shunt resistance is decreased the voltage decays more rapidly. The base case has a shunt resistance of 160 MΩ, the kink at 50 ms is caused by this parallel resistance. The voltage decay before 50 ms shows a logarithmic dependence on time similar as observed by Elliott and co-workers [58]. In the case of deep traps the decay rate is higher as visible in Figure 8(c). With shallow traps the voltage decay is slower as charges are immobilized when trapped delaying the recombination. In perovskite solar cells, a persistent photovoltage was observed after light turn-off [59] that might be caused by mobile ions.

The open-circuit voltage *V*_oc_ in a solar cell can be described according to

(10) $$V_{\text{oc}}=\frac{E_{g}}{q}-\frac{k_{B}\cdot T}{q}\cdot \ln\left(\frac{N_{0}^{2}}{n\cdot p}\right),$$

where *E*_*g*_ is the energy of the band-gap, *q* is the unit charge, *k*_*B*_ is the Boltzmann constant, *T* is the temperature, *N*_0_ is the effective density of states, *n* the electron density and *p* the hole density. When the decay of a homogeneous charge carrier density (d*n*/d*t* = −*β*⋅*n* ^2^ with *n* = *p*) is inserted into Equation (10) we obtain

(11) $$V_{\text{ocvd}}\left(t\right)=\frac{E_{g}}{q}-2\cdot \frac{k_{B}\cdot T}{q}\cdot \ln\left(N_{0}\cdot \left(\frac{1}{n\left(0\right)}+\beta \cdot t\right)\right),$$

where *n*(0) is the initial charge carrier density at open-circuit and *β* is the recombination pre-factor. According to Equation (11), the voltage signal is expected to decay with a logarithmic dependence on time. This is shown in the plots of Figure 8 as grey lines. Parameter *β* is chosen according to the ‘base’ case. The analytic solution (Equation (11)) does only fit the numerical simulation at the very beginning. The reason is that the charge is not homogeneously distributed inside the device [60]. Close to the electrodes the densities are higher and charges flow slowly into the middle of the device where they recombine. Zero-dimensional models are therefore not suited to describe the open-circuit voltage decay in p-i-n structured solar cells. The same consideration also applies to recombination coefficients extracted from CELIV using the OTRACE method or to lifetimes determined from TPV or IMVS which are also described in this manuscript.

From OCVD measurements no material parameters can be derived directly. It can, however, be useful for comparing different devices or to perform parameter extraction by fitting numerical simulations (see last section).

#### 3.4.1. Transient photovoltage and charge carrier lifetime

Transient Photovoltage (small-signal TPV) is frequently performed to determine charge carrier lifetimes in organic solar cells [13,37,57,61–63]. The concept of ‘charge carrier lifetimes’ stems from the community of silicon solar cells and describes how long on average a minority charge carrier survives in a doped bulk material [1]. A general definition of minority charge carrier lifetime *τ* is

(12) $$\tau =\frac{n}{R},$$

where *n* is the charge carrier density (electrons or holes) and *R* is the recombination current. In a device with a high and homogeneous doping density (majority charge carrier) the minority charge carrier has a lifetime that is constant in space and time.

In p-i-n structures, the charge carriers are generated in the intrinsic region and transported to the electron and hole contact layers. The intrinsic region has no doping and consequently also no clear majority or minority carriers. Both electron and hole densities vary spatially even at open-circuit [60]. The charge carrier lifetime is therefore not clearly defined in a p-i-n structure and it is position-dependent. Physical conclusions based on measured ‘charge carrier lifetimes’ can therefore be misleading. Despite these limitations lifetimes are often determined also for thin p-i-n structured devices [13,37,57,61–63]. In the supplemental information, we present detailed simulation results of TPV lifetimes and compare them with the theoretical lifetime. Thereby the simulated lifetimes only match the theoretical lifetime in a few cases. This is consistent with the findings of Kiermasch et al. [64] stating that in thin devices often capacitive discharge is measured instead of a bulk charge carrier lifetime. In general, the agreement is better at high light intensities since the charge carrier distributions are more uniform. We recommend to interpret measured charge carrier lifetimes from p-i-n structures carefully. In thick devices, the problem is less severe as the charge carrier gradients are smaller [60].

In a TPV experiment, the solar cell is kept at open-circuit voltage under bias-illumination. Then an additional small laser pulse (or LED pulse) is applied to the device to create some additional charge that decays exponentially thereafter. If the light pulse is small enough the assumption that the change in density of photogenerated carriers is proportional to the photovoltage increase (Δ*n* ~ Δ*V*) holds. The voltage decays as

(13) $$V\left(t\right)=V_{\text{oc}}+\Delta V\cdot \text{exp}\left(-t/\tau \right),$$

where *V*_oc_ is the open-circuit voltage at the bias illumination, Δ*V* is the voltage increase due to the laser pulse and *τ* is the minority carrier lifetime. By the TPV experiment, the charge carrier lifetime at given bias illumination can be estimated directly from the exponential voltage decay. Charge carrier lifetimes are usually plotted versus the charge carrier density.

Lifetimes from TPV are not a direct measure of the steady-state charge carrier lifetime as shown by O’Regan et al. [65]. To obtain steady-state carrier lifetimes, the TPV lifetimes need to be multiplied with the reaction order (often denoted as *λ* + 1) [57,65].

In the supplemental material, we show TPV simulation and analysis with lifetime calculation on the basis of our defined cases. Lifetime values can be calculated but their interpretation is difficult, since the underlying assumptions do not hold. We recommend to interpret TPV lifetimes on p-i-n structured devices very carefully.

### 3.5. Deep level transient spectroscopy

Deep level transient spectroscopy (DLTS) is a technique that was developed to study trapping in semiconductor devices. In DLTS a capacitance, a current (i-DLTS) or charge (Q-DLTS) is measured over time after the application of a voltage step at various temperatures. DLTS was introduced by Lang in 1974 measuring capacitance transients of GaAs semiconductor devices at varied temperature [66]. The technique promises to determine trap spectra (trap density versus energetic trap depth) of majority and minority carrier traps as well as capture cross-sections. It is frequently applied to study defect distributions in inorganic semiconductors [66–70]. DLTS is of limited use for organic semiconductors since their mobility is too low and RC-effects are usually too high [71].

Great care must be taken to accurately determine trap spectra in organic or quantum dot semiconductors. When measuring capacitance-based DLTS the probing frequency must be small enough to measure the space-charge capacitance [71]. When measuring current-based DLTS it is important to properly subtract the displacement current [72] and measure with high current resolution [73]. DLTS has also been performed on perovskite solar cells to determine trap energies and densities [74]. Such results should however be carefully interpreted as the presence of mobile ions may disturb the measurement.

In this review, we simulate current-based DLTS [68,72,73,75]. A negative voltage step (0 to −5 V) is applied to the device in the dark and the transient current response is analysed. Apart from the displacement current caused by RC-effects there is a small current from trap emission. The trap emission current *j*_te_ from a discrete energy trap can be described as

(14) $$j_{\text{te}}\left(t\right)=\frac{1}{\tau_{\text{te}}}\cdot q\cdot d\cdot N_{t}\cdot \exp\left(-\frac{t}{\tau_{\text{te}}}\right),$$

where *τ*_te_ is the trap emission time constant, *q* is the unit charge, *d* the device thickness (or depletion width in thick devices) and *N*_*t*_ is the trap volume density. The trap emission time *τ*_te_ is the inverse of the trap emission rate *e*_*t*_ and is described as

(15) $$\tau_{\text{te}}=\frac{1}{e_{t}}=\frac{1}{c_{t}\cdot N_{0}}\cdot \exp\left(\frac{\Delta E}{k_{B}\cdot T}\right),$$

where *c*_*t*_ is the trap capture rate, *N*_0_ is the number of chargeable sites (density of states), Δ*E* the trap depth, *k*_*B*_ the Boltzmann constant and *T* the temperature. The trap capture rate *c*_*t*_ can be considered as material constant that includes the capture cross-section. For inorganic semiconductors the trap emission time includes another factor 1/*T* ^2^ to account for the temperature dependence of the thermal velocity and the temperature dependence of the density of states [70].

We distinguish between two distinct shapes of the current decay from thermal emission of trapped carriers. The emission current from single energy trap levels (Equation (14)) is exponentially decaying. The emission current from an exponential band tail shows a power-law decay.

Street analysed current decays after illumination turn-off with thermal emission of carriers from exponential band tails [76]. Such a TPC current decay is consistent with the DLTS current decay after the transit time. The emission current *j*_em_ from the exponential band tail *N*(*E*) = *N*_*D*_⋅exp(−*E*/*E*_0_) is described as

(16) $$j_{\text{em}}\left(t\right)=q\cdot d\cdot N_{D}\cdot k_{B}\cdot T\cdot \omega^{-\frac{k_{B}\cdot T}{E_{0}}}\cdot t^{\left(-\frac{k_{B}\cdot T}{E_{0}}-1\right)},$$

where *N*(*E*) is the density of states as a function of energy, *N*_*D*_ is the density at 0 eV with unit cm^−3^ eV^−1^, E is the energy from the band edge (*E* = 0) into the band-gap, *E*_0_ is the band tail slope, *q* the unit charge, d the device thickness, *k*_*B*_ the Boltzmann constant, *T* the temperature and *ω* is the attempt-to-escape factor (on the order of 10^12^ 1/s) [76].

**Figure 9.**
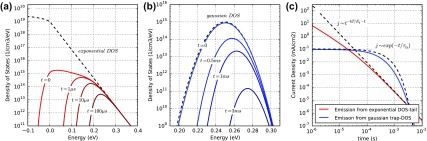
Calculation of the thermal emission of charge carriers from the density of states. (a) The dashed line is the density of states with square-root dependence above the band edge and exponential dependence inside the band. The solid lines represent the charge carrier distributions at different times. The LUMO-level is located at 0 eV, positive energy values reach into the band-gap. (b) Same as in (a) but for a Gaussian DOS. (c) Calculated currents from carrier emission of (a) and (b) including analytical fits according to Equations (14) and (16).

To illustrate the different current decay shapes, we calculate the emission current from two different densities of states. First the density of states is filled with charges using Fermi–Dirac-statistics, then the emission current over time is calculated. Charge transport inside the device is neglected. In Figure 9 the carrier distribution and emission current from an exponential trap-DOS and a Gaussian trap-DOS are shown. The initial Fermi-level was chosen as 0.2 eV. The traps DOS in Figure 9(b) is therefore completely filled. The exponential tail is filled below 0.2 eV. The emission current over time from the exponential DOS follows a power-law decay (Figure 9(c)) and is described well for longer times using Equation (16). The emission from the Gaussian trap DOS is exponential and reflected by Equation (14). In reality, a combination of both may be observed. Furthermore, emission currents from both electrons and holes will make the analysis more difficult. For simplicity we use single energy traps and discrete band energies for the simulations of DLTS below.

**Figure 10.**
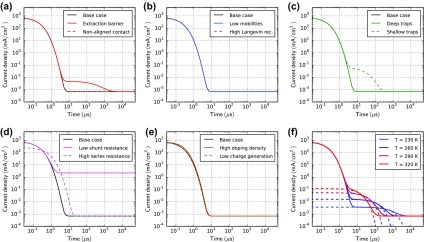
DLTS simulations for all cases in Table 1. The voltage is 0 V for *t* < 0. At *t* = 0 the voltage jumps to −5 V. (f) DLTS simulations of case ‘shallow traps’ at different temperatures (solid lines). The dashed lines are exponential fits according to Equation (14).

Figure 10 shows DLTS simulations at room temperature. In contrast to the results of the rate equation model in Figure 9(c), the results in Figure 10 were obtained with the drift-diffusion software Setfos [21] that considers the position-dependence of carrier transport in the device. The current peak within the first 1 μs is caused by RC-effects and is not of interest here. The recombination pre-factor and the mobility have no influence on the resulting current (Figure 10(b)). For ‘shallow traps’ an additional current flow from trap emission is observed (Figure 10(c)). The deep traps lead to SRH-recombination – trapped charges recombine instead of being re-emitted. An extraction barrier as shown in Figure 10(a) can however lead to a current tail that might be mistaken for trap emission. When the device has a low shunt resistance as shown in Figure 10(d) the trap emission current is hidden by the leakage current through the shunt. If the device is doped some of the equilibrium charge is extracted that leads to an additional current (Figure 10(e)).

Figure 10(f) shows the simulation results of case ‘shallow traps’ at different temperatures. The dashed lines represent exponential fits using Equation (14). Using the extracted trap emission time *τ*_te_, the trap-depth can be calculated using Equation (15) in an Arrhenius plot. The trap depth of 0.4 eV can be accurately determined when analysing the simulation results and is thus consistent with this model input parameter. For the number of occupied traps values between 7⋅10^14^ and 1.6⋅10^15 1^/cm^3^ were extracted. The effective density of occupied traps at room temperature in the dark is 2⋅10^16 1^/cm^3^ for the case ‘shallow traps’. The analytical fit from the emission current thus underestimates the trap density by a factor of 10 in this case. The reason is that also at −5 V not all the traps are empty. The effective trap density is therefore likely to be underestimated with this method.

In our simulation, there is no limit for the current resolution. In measurements it can however be difficult to resolve 6 orders of magnitude of currents in this time-regime. Trap emission might be hidden in measurement noise.

### 3.6. Transient photocurrent

In transient photocurrent (TPC) experiments the current response to a light step is measured at constant offset-voltage. The current rise and decay reveal information about the charge carrier mobilities, trapping and doping. TPC is usually performed with varied offset-voltage, offset-light or light pulse intensity. The rise time in organic solar cells usually lies between 1 and 100 μs. In perovskite solar cells, the current rise starts in the microsecond regime and can take several seconds until steady-state is reached [24].

Christopher McNeill and co-workers observed a photocurrent overshoot in polymer solar cells and explained it by charge trapping and detrapping using drift-diffusion simulations [77]. If the charge trapping is slow enough, it leads to a current overshoot caused by space charge effects. As more and more charges get trapped they screen the electric field and hinder charge transport. Fast trapping however leads to a slower current rise [78]. In some cases, a current overshoot occurs only at negative bias voltage [61].

The current decay can be described in the same manner as in DLTS. Using Equation (14) trap emission currents from discrete energies can be calculated. Using Equation (16) trap emission from an exponential DOS tail is calculated. Street calculated the density of states of the band tail of PCDTBT:PCBM and P3HT:PCBM solar cells by analysing the TPC current decay [76].

By integrating the current decay over time, the extracted charge is obtained [77]. In our simulations, the extracted charge is one or two orders of magnitude lower than the effective charge inside the device. During extraction most of the charge recombines. The fraction depends on the relative time scale of recombination with respect to charge extraction.

**Figure 11.**
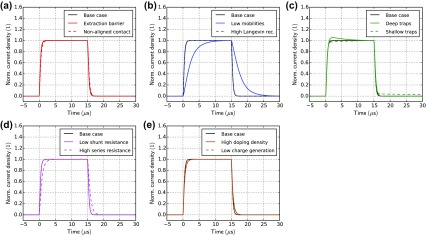
Transient photocurrent simulations for all cases in Table 1. At *t* = 0 the illumination is turned on. At *t* = 15 μs the illumination is turned off. The applied voltage is 0 V. The current is normalized by the current at 15 μs.

Figure 11 shows TPC simulations with light pulses of 15 μs duration. The shape of the current rise does not change for the cases: ‘extraction barrier’ (a), ‘non-aligned contact’ (a), ‘high Langevin recombination’ (b), ‘low shunt resistance’ (d) and ‘low charge generation’ (e). A smaller charge carrier mobility clearly leads to a slower rise and decay as shown in Figure 11(b). The shallow traps fill slowly (capture and re-emission) and lead to a slower equilibration of the current (c). The trap emission leads to a slow exponential current decay after light turn-off. The case with deep traps shows a current overshoot (c) consistent with the analysis of McNeill [77]. Space-charge is built up by the charged traps reducing the current on a longer timescale. If TPC is performed with offset-light, the current overshoot and the long decay vanish because the offset-light keeps the traps filled [77]. In our simulations, this effect is already visible with offset-light intensities of 0.1% of the pulse illumination intensity. A high series resistance can also lead to a slower current rise and decay as shown in Figure 11(d). The case ‘high doping density’ shows a slightly longer current rise and decay caused by space charge effects. With imbalanced mobilities, two time-constants arise corresponding to the fast and the slow carrier type as shown in Figure S8 in the SI.

In contrast to CELIV there is no simple formula to extract the charge carrier mobility from TPC data. TPC is however a powerful technique to study charge transport, identify trapping and to extract parameters using numerical modelling.

### 3.7. Charge extraction

Charge extraction (CE) was introduced by Duffy et al. [79] in 2000 to measure the charge carrier density in dye-sensitized solar cells. It was applied to organic solar cells by Shuttle et al. [80] and is frequently utilized to measure charge carrier density at varied light intensity [37,57,62,81]. It is sometimes also referred to as photo-induced charge extraction (PICE) or time-resolved charge extraction (TRCE) [57]. When a negative extraction voltage is used it is referred to as bias amplified charge extraction (BACE) [82].

In the charge extraction experiment the solar cell is illuminated and the open-circuit voltage is applied such that no current flows (*V*_oc_). In this state, all charge carriers generated by light recombine. At *t* = 0 the light is switched off and simultaneously the voltage is set to zero (or reverse bias [82,83]). The charge carriers are extracted by the built-in field and lead to a current. Integrating the extraction current over time yields the extracted charge. The charge carrier density *n*_CE_ is then calculated according to

(17) $$n_{\text{CE}}=\frac{1}{d\cdot q}\cdot \left(\int_{0}^{t_{e}}j\left(t\right)\cdot dt-\left(V_{a}-V_{e}\right)\cdot C_{\text{geom}}\right),$$

where *d* is the device thickness, *q* is the unit charge, *t*_*e*_ is the extraction time (usually 1 ms is enough), *j*(*t*) is the transient current density, *C*_geom_ is the geometric capacitance, *V*_*a*_ the voltage applied prior extraction (in most cases *V*_oc_) and *V*_*e*_ is the extraction voltage. The charge on the capacitance needs to be subtracted [83] because only the charge carrier density inside the bulk is of interest.

When the experiment is performed with varied delay time between light turn-off and charge extraction, CE can also be used to study recombination [57,79,83]. The technique is then very similar to CELIV with OTRACE [44] described in the section above.

**Figure 12.**
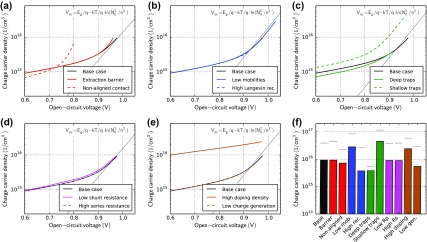
Charge extraction simulations for varied light intensity (and thus *V*_oc_) for all cases defined in Table 1. The current is integrated over time according to Equation (17) to obtain the charge carrier density (the charge on the capacitance is subtracted). The light intensity is varied by five orders of magnitude. The grey-line is the theoretical *V*_oc_ for *n* = *p* in a zero-dimensional model. (f) Extracted charge carrier density at the highest light intensity. Grey lines represent the effective amount of photogenerated charge at open-circuit obtained from the simulated charge carrier profiles.

Figure 12 shows simulation results of charge extraction for all cases with varied light intensity. Changing the mobility or the recombination pre-factor changes, the open-circuit voltage *V*_oc_ but has no major influence on the relation charge carrier density versus the *V*_oc_ (b). The thin grey line is the theoretical open-circuit voltage from a zero dimensional model assuming equal electron and hole densities. At higher light intensity the trend agrees well with the simple model. At low light intensity the zero-dimensional model fails due to stronger spatial separation of electrons and holes.

The case ‘deep traps’ (c) has a similar *n* vs. *V*_oc_ curve. The ‘shallow traps’ (c), however, lead to a higher density of extracted charges. Trapped charge carriers are ‘protected’ from recombination. Therefore, a higher charge density can accumulate at *V*_oc_. The *V*_oc_ in the case ‘non-aligned contact’ (a) is lower. More charge is required to reach the same *V*_oc_. It is far away from the ideal curve shown in grey. The series resistance (d) has no influence on the extracted charge. The extraction current is slowed down, but the current-integral remains constant. Interestingly, the charge carrier density is much higher in the case ‘high doping density’. The device is p-doped, so there are less electrons under illumination compared to the un-doped case. Under illumination, the depletion region gets smaller and more holes can accumulate compared to the un-doped case.

In Figure 12(f), the extracted charge at the highest light intensity is compared to the effective photogenerated charge in the device at open-circuit. The extracted charge carrier density is in all cases lower than the effective charge carrier density at open-circuit. In our simulations, between 15 and 70% of the charge is extracted (see grey line in Figure 12(f)). Applying a negative extraction voltage *V*_*e*_ reduces recombination losses [82,83]. Indeed, in our simulations more charge is extracted (between 20 and 90% at −3 V) using a negative extraction voltage.

Our case study is based on a device with a rather high Langevin recombination efficiency of 0.1. If Langevin recombination is turned down to 10^−3^ in our simulation more than 90% of the charge is indeed extracted. The accuracy of the charge extraction results therefore critically depends on the recombination.

### 3.8. Impedance spectroscopy

Impedance spectroscopy is a popular technique to investigate solar cells. It is abbreviated as IS or EIS (electro-chemical impedance spectroscopy). It is also called admittance spectroscopy (admittance is the inverse of the impedance). In impedance spectroscopy, a small sinusoidal voltage *V*(*t*) is applied to the solar cell according to

(18) $$V\left(t\right)=V_{0}+V_{\text{amp}}\cdot \sin\left(\omega \cdot t\right),$$

where *V*_0_ is the offset voltage, *V*_amp_ is the voltage amplitude and *ω* is the angular frequency 2⋅*π*⋅*f*. If the voltage amplitude *V*_amp_ is small enough the system can be considered as linear therefore the current density *j*(*t*) is also sinusoidal. The amplitude and the phase-shift of the current are analysed. Impedance spectroscopy is performed at various frequencies and/or offset voltages (see next section) and/or offset illuminations. Using the transient voltage and the transient current signal the complex impedance *Z* is calculated according to

(19) $$Z=\frac{1}{Y}=\frac{\int_{0}^{N\cdot T}V\left(t\right)\cdot \exp\left(i\cdot \omega \cdot t\right)\cdot dt}{\int_{0}^{N\cdot T}j\left(t\right)\cdot \exp\left(i\cdot \omega \cdot t\right)\cdot dt},$$

where *Y* is the admittance, *N* is the number of periods, *T* is the period 1/*f*, *i* is the imaginary unit and *ω* is the angular frequency. For the analysis of the impedance, often the capacitance *C* and the conductance *G* are plotted versus frequency or offset voltage are calculated according to

(20) $$C=\frac{1}{\omega }\cdot \text{Im}\left(\frac{1}{Z}\right)$$

and

(21) $$G=\text{Re}\left(\frac{1}{Z}\right),$$

where *ω* is the angular frequency, Im() denotes the imaginary part and Re() the real part.

Usually, impedance spectroscopy data is plotted in the so-called Cole-Cole plot. Here, the real and imaginary part of the impedance *Z* is plotted in the complex plane for the different frequencies. We show such simulation results in the supplemental information. Alternatively, the capacitance *C* is plotted versus the frequency.

One of the main advantages using impedance spectroscopy is that effects occurring on different timescales can be separated. Trapping and de-trapping for example occurs usually on longer time scale (lower frequency) compared to transport of free carriers. Most commonly impedance spectroscopy data is analysed using equivalent circuits. Thereby electric circuits are constructed from resistors, capacitors, inductors and further electric elements such that the measured frequency-dependent impedance can be reproduced [84–88]. The disadvantage of equivalent circuits is that the results can be ambiguous and the parameters cannot be directly associated with macroscopic material parameters.

Knapp and Ruhstaller solved the drift-diffusion equations with a small signal analysis to simulate impedance spectroscopy data [89,90]. Here, physical parameters are used as simulation input that allow direct interpretation of the results. The same approach is implemented in the software Setfos [21] that we apply in this study.

Measuring the capacitance is a way to probe the occupation of trap sites due to space charge effects [91]. Slow traps can increase the capacitance at low frequencies as shown by numerical simulation [89,90]. Also slow ionic charges which might be present in perovskite solar cells can lead to an increase of the capacitance at low frequencies [50,87]. Recombination of charge carriers leads to a decrease in the capacitance – it can even become negative. Also self-heating of a device can lead to a negative capacitance as analysed by Knapp and Ruhstaller [92]. A positive capacitance means that the phase-shift between voltage and current is positive (voltage leading the current), a negative capacitance means that the phase-shift becomes positive (current leading the voltage).

In the SI, we show impedance simulations under illumination with varied offset-voltage plotted in the Cole–Cole representation. It is often argued that the size of the semi-circle in the Cole–Cole plot represents the recombination in the device. From our simulation results, we conclude that many effects influence the size of the semi-circle in the complex plane. We, therefore, advise to interpret such results carefully.

The real part of the impedance at low frequency coincides with the inverse of the current slope in the JV-curve at the same offset-voltage. If the probing frequency is low enough one basically measures the DC properties. Thus, an IV-curve can be used as consistency check of the impedance measurement. From low-frequency impedance data, the JV-curve can be reconstructed without using equivalent circuits [84].

**Figure 13.**
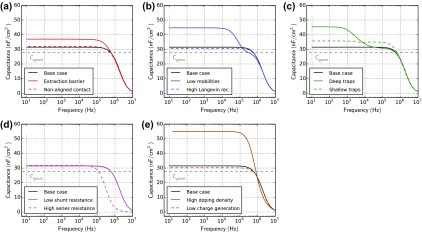
Impedance simulations for all cases in Table 1. The capacitance *C* is calculated according to Equation (20). The offset-voltage is 0 and offset-light is turned on. The dashed grey line represents the geometric capacitance.

Figure 13 shows impedance simulations of all cases. In the base case mainly RC-effects are observed. Due to the background illumination the capacitance is however slightly higher than the geometric capacitance of 27 nF/cm^2^. A large amount of charge in the bulk leads to a reduced depletion region – and consequently to a higher capacitance. The extraction barrier (a), the low mobility (b), traps (c) or doping (e), therefore, lead to an increase in the capacitance under illumination. In the case of deep and slow traps (c), this capacitance rise occurs only at low frequency. If the probing frequency is too high, charges cannot be trapped and de-trapped during one period. These slow traps are therefore invisible at high frequencies (for example 100 kHz in plot Figure 13(c)). With shallow traps the de-trapping is much faster – therefore the capacitance-rise happens already at faster timescale.

In all cases, the capacitance decreases at frequencies above 1 MHz due to RC-effects. In the case with a higher series resistance (d), the capacitance decrease shifts to lower frequencies due to a higher RC-time. The impedance of the RC-effects *Z*_RC_ can be calculated according to

(22) $$Z_{\text{RC}}\left(\omega \right)=R_{S}+\frac{1}{i\cdot \omega \cdot C_{\text{geom}}},$$

where *R*_*S*_ is the series resistance, *i* the imaginary unit, *ω* the angular frequency and *C*_geom_ the geometric capacitance. Using Equation (22) the series resistance and the geometric capacitance can be determined from a capacitance-frequency plot in the dark.

#### 3.8.1. Capacitance-voltage

In capacitance-voltage (CV) measurements the impedance is measured at constant frequency and the offset-voltage is varied. The capacitance is calculated according to Equation (20). To measure CV usually frequencies below 50 kHz are used. In most diode-like devices, CV shows a peak at forward voltage. The position of this peak is usually independent of the probing frequency and independent of the device thickness [93]. The peak voltage is usually smaller than the built-in voltage [94] and it can be regarded as an effective value for the conduction onset [95]. The height and the voltage of the capacitance-peak is related to carrier injection [96] (the injection barriers and the built-in voltage). In bipolar devices like solar cells, the capacitance-peak cannot be directly related with an analytical expression as shown for unipolar devices [94].

The increase in the capacitance is caused by a space-charge effect. When the voltage increases charges are injected and the depletion width decreases – leading to an increase in capacitance. After a certain voltage conduction starts and the capacitance decreases again and can even get negative. Negative capacitances can be caused by recombination or self-heating [92].

CV can be used to monitor the change of injection barrier for example during degradation [10,11,97,98]. In bilayer devices, CV can result in a plateau instead of a peak as observed for Alq_3_/NPB devices [8,98]. At a certain voltage charge carriers are injected into one of the two layers. When one layer is flooded with carriers only the ‘parallel plate’ capacitance of the remaining layers is observed leading to a higher capacitance plateau until charges are injected into the second layer as well. The effect is observable as long as the injection into the two layers occurs at different voltages. Materials with a permanent dipole moment facilitate different electron and hole injection voltages in bilayer devices. Using CV, the macroscopic polar sheet charge of such materials can be determined [99].

**Figure 14.**
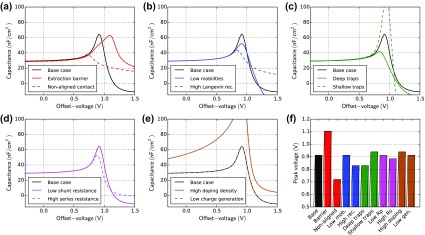
Capacitance-voltage simulations for all cases in Table 1 without offset illumination. The capacitance *C* is calculated according to Equation (20). The frequency is kept constant at 10 kHz. (f) Voltage where the capacitance reaches a maximum.

Figure 14 shows CV simulations of all cases. Significant changes in the peak voltage are only observed in the cases where the charge injection is changed. The case ‘non-aligned contact’ (a) has a lower built-in voltage which leads to a decrease of the peak-voltage. The case ‘extraction barrier’ (a) has the same built-in voltage but an additional barrier to overcome and thus the CV peak is shifted to higher voltages. In all other cases, only a slight change in CV peak voltage is observed. CV seems therefore suited to investigate charge injection and the built-in voltage.

#### 3.8.2. Mott–Schottky analysis of capacitance-voltage measurements

Mott–Schottky analysis is a popular technique applied to CV data to extract the doping density and the built-in voltage using the relation

(23) $$\frac{1}{C^{2}}=\frac{2}{S^{2}\cdot \varepsilon \cdot q}\cdot \frac{1}{N_{A}}\cdot \left(V_{\text{bi}}-V\right),$$

where *C* is the capacitance, *S* is the device area, *ε* is the permittivity, *q* is the unit charge, *N*_*A*_ is the doping density in the bulk and *V*_bi_ is the built-in voltage. The quantity 1/*C* ^2^ is linear with the voltage and allows one to determine the doping density *N*_*A*_ and the built-in voltage. It has however been shown that the analysis returns erroneous results in thin semiconductors. Kirchartz et al. simulated an un-doped device with 100 nm thickness. The Mott–Schottky analysis of the simulated CV data resulted in an apparent doping density of 1⋅10^16 1^/cm^3^ even though no doping was assumed in the simulation [100] – a clear indication that the technique should not be used for thin semiconductor layers like organic solar cells.

Tripathi and Mohapatra proposed to use the relation 1/C^2/3^ for the analysis of organic devices [93]. Their analysis is however based on the assumption of a unipolar device and is therefore also not suited for the analysis of solar cells. We propose to use dark-CELIV to estimate the lower limit of the doping density of organic solar cells.

The determination of the built-in potential with a Mott–Schottky analysis is also erroneous as shown by Mingebach et al. [101]. Mott–Schottky analysis should only be performed on devices that are thick enough and highly doped.

### 3.9. Intensity-modulated photocurrent spectroscopy

In intensity-modulated photocurrent spectroscopy (IMPS) the device is illuminated with a modulated light intensity and the photocurrent is measured. The voltage is kept constant. The modulated light intensity *L*(*t*) is described as

(24) $$L\left(t\right)=L_{0}+L_{\text{amp}}\cdot \sin\left(\omega \cdot t\right),$$

where *L*_0_ is the offset light intensity, *L*_amp_ is the amplitude of the modulation (typically 5–10% of *L*_0_) and *ω* is the angular frequency 2⋅*π*⋅*f*. Like in impedance spectroscopy the theory for IMPS is based on the linearization of the device at a working point, which is valid as long as the light intensity amplitude *L*_amp_ is small enough. In this case, also the current is sinusoidal and the phase shift and amplitude are studied. The complex IMPS quantity *Z*_IMPS_ is calculated according to

(25) $$Z_{\text{IMPS}}=\frac{\int_{0}^{N\cdot T}j\left(t\right)\cdot \exp\left(i\cdot \omega \cdot t\right)\cdot dt}{\int_{0}^{N\cdot T}L\left(t\right)\cdot \exp\left(i\cdot \omega \cdot t\right)\cdot dt},$$

where *N* is the number of periods, *T* is the period 1/*f*, *i* is the imaginary unit and *ω* is the angular frequency. The concept and analysis of IMPS are similar to impedance spectroscopy – in impedance spectroscopy the voltage is modulated and in IMPS the light is modulated.

In 1985, the first IMPS theory was introduced by Li and Peter to describe semiconductor–electrolyte interfaces [102]. It was later refined and frequently used to characterize dye-sensitized solar cells (DSSC) [103–106]. For the analysis of IMPS data a transport time-constant *τ*_tr_ is calculated according to

(26) $$\tau_{\text{tr}}=\frac{1}{2\cdot \pi \cdot f_{\text{peak}}},$$

where *f*_peak_ is the frequency where the imaginary part of the IMPS quantity reaches a maximum. In dye-sensitized solar cells, the electron diffusion coefficient is calculated from the transport time-constant (*D*_*n*_ = *d* ^2^/(2.35⋅*τ*_tr_)) [105]. In DSSC there is the common assumption of a fully screened electric field by the ionic charge of the electrolyte. Therefore, electron diffusion dominates transport and can be characterized by IMPS. In organic and other third-generation solar cells this assumption does not hold. In this case there is no mathematical framework available yet for the analysis of IMPS measurements. In degraded organic solar cells, a negative phase shift was observed at certain frequency ranges – meaning that the current leads the illumination. Set et al. used drift-diffusion simulations to show that negative IMPS phase shifts are caused by trap-assisted recombination [107]. Indeed, in our model, we observe minor negative phase shifts only for the case with deep traps at low light intensity. At low frequency the real part of the IMPS signal equals the steady-state photocurrent [106].

IMPS has also been applied as imaging technique to study morphological phases in bulk-heterojunction solar cells [108]. In perovskite solar cells, a second peak at 10 Hz was observed and attributed to ionic motion [109].

**Figure 15.**
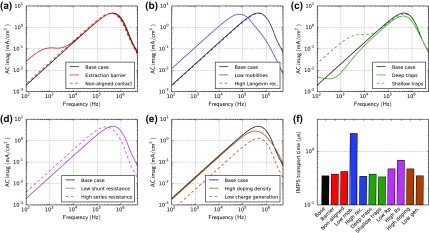
IMPS simulations for all cases in Table 1 with low offset light intensity (3.6 mW/cm^2^). The offset voltage is zero. (f) IMPS transport time-constant calculated according to Equation (26).

Figure 15 shows the imaginary part of the IMPS simulations for all cases. In all cases a peak at high frequency is observed. It can be related to charge transport – only the case ‘low mobilities’ (b) leads to a significantly longer transport time-constant and thus the peak shifts to lower frequency. Trapping and de-trapping (c) as well as an extraction barrier (a) can lead to an additional peak/shoulder at low frequency. The series resistance slows down charge transport (d) as in all transient experiments, thus shifting the peak to lower frequency. All other cases show no distinct features.

In certain measurements, two peaks in IMPS are observed. If the electron and hole mobilities are imbalanced, two peaks can arise as we show in the SI.

#### 3.9.1. Intensity-modulated photovoltage spectroscopy

In intensity-modulated photovoltage spectroscopy (IMVS), the device is kept at open-circuit and the photovoltage is measured. IMPS and IMVS are closely related. In IMPS, the voltage is constant and the sinusoidal current is measured. In IMVS the current is zero and the sinusoidal voltage is measured.

Classically, from IMVS measurements the charge carrier lifetime is extracted using the frequency where the imaginary part reaches a minimum [106,110,111]. As outlined in the section on transient photovoltage, the quantity charge carrier lifetime is not physically meaningful in p-i-n structured devices. Our simulation results show that at open-circuit, the device behaviour is not only governed by recombination (as commonly expected) but also by charge transport, which is in line with findings of Street [76]. Up to now there is no straight-forward interpretation of IMVS measurement results. We show IMVS simulations of all cases in the SI. Our simulation results show that charge carrier lifetimes extracted from IMVS and TPV are fully consistent.

### 3.10. Further characterization techniques

There are a number of further opto-electrical characterization techniques for solar cells that we describe here only briefly without being exhaustive.

*Displacement current measurement (DCM)* is a technique that is used to study the capacitance of multi-layered devices and estimate trap densities [97]. In DCM, a triangular voltage is applied to the device in the dark in two cycles. Compared to CELIV in DCM the voltage ramp goes up and down such that both the injection and the extraction of carriers can be studied. When carriers are injected into one layer the capacitance of the multilayer system changes and so does the displacement current. Comparing the first and the second cycle allows one to estimate the trap density.

In *dark injection transients (DIT),* a voltage step is applied to a device and the transient current is measured. The device under investigation needs to be unipolar (only one charge carrier type can be injected) and good Ohmic contacts are required. A space-charge effect leads to a current overshoot. Therefore, this technique is also called transient space-charge-limited current (T-SCLC) in the literature. The time of the current overshoot is related to a transit time and allows the estimation of the charge carrier mobility and its field dependence [112,113]. The occurrence of the current overshoot is a confirmation of good electrical contact for charge injection.

In *double injection transients (DoI)* a voltage step is applied to the device. Compared to dark injection transients this technique is applied to ambipolar devices where electrons and holes can be injected. It leads to a slow current rise until steady-state is reached. The electron mobility, hole mobility and recombination pre-factor determine the current rise dynamics and can be estimated by formulas [112,114]. We show simulation results of DoI in the SI.

*Open*-*circuit voltage* versus *temperature*: The open-circuit voltage is measured at varied temperature to estimate the built-in voltage and the band-gap [101]. We have simulated open-circuit voltages versus temperature and found that the built-in voltage is extracted accurately from *V*_oc_ at low temperature. With extrapolation to zero Kelvin the band-gap can be extracted accurately except for the cases ‘non-aligned contact’ and ‘extraction barrier’. The simulation results can be found in the SI.

*Differential charging* combines small-perturbation transient photocurrent (TPC) and transient photovoltage (TPV) measurements. From the two experiments the differential capacitance *C* = Δ*Q*/Δ*V* is calculated for varied light intensity. The integral reveals the charge carrier density at open-circuit [13,63]. The charge Δ*Q* stems from the current-integral of TPC whereas the Δ*V* is the change in voltage in TPV. Both experiments are performed with offset-light and a small light pulse.

In *time*-*delayed collection field (TDCF)* the device is kept at a constant voltage when a short laser pulse is applied [115,116]. After a delay-time, a reverse bias is applied to extract the charge carriers. TDCF can be used to investigate the field dependence of charge generation and recombination. A low RC-time is required for this experiment.

*Thermally stimulated current (TSC)* is a technique to measure trap spectra in semiconductors. The device is illuminated and cooled down to very low temperatures (<50 K). Then the illumination is turned off and the device is slowly heated back to room temperature. The current resulting from trap emission is measured over time. Shallow traps are released at low temperatures and deeper traps are released at higher temperature. Trap density and trap energy levels can be estimated [117].

In *thermal admittance spectroscopy (TAS)*, impedance spectroscopy is measured at different temperature levels. Similar to DLTS full trap spectra can be extracted analysing the capacitance-frequency relation [91]. It is also possible to determine activation energies for mobility and injection [49].

*Transient absorption spectroscopy (TAS)*: This technique takes advantage of the fact that in some materials infrared light is absorbed by free charge carriers. The device is illuminated by infrared light (usually at a wavelength around 1000 nm) and the transmitted or reflected light is measured with a photo-detector. An additional optical light pulse creates charge carriers that are then monitored over time by the infrared light to investigate recombination dynamics [57,63,65].

*Time*-*of*-*flight (TOF)* is a technique to measure the charge carrier mobility in semiconductors [112,114,118]. A short laser pulse generates a small amount of charge carriers on one side of the semiconductor layer. Due to an applied voltage the charge carrier package drifts through the layer. From the transit time, the mobility is calculated. The advantage of the technique is that electron and hole mobilities can be measured separately. A disadvantage is that the technique requires thick samples (>1 μm) and blocking contacts. Therefore, it cannot easily be applied to regularly prepared solar cells [112].

## 4. Comprehensive parameter extraction with numerical simulation

In the previous sections, we presented an overview over various measurement techniques for solar cells. Their interpretation allows mainly qualitative conclusions: devices can be compared and trends can be observed. When monitoring device ageing conclusions can be drawn regarding the physical origin of the degradation [10,11,19].

Organic and other third-generation solar cells are devices with complex charge transport physics. Simple analytical device descriptions are often not capable to capture all relevant physical effects. Parameters cannot easily be determined by simple methods. The analysis with analytical expressions as for photo-CELIV or for Mott–Schottky can lead to inaccurate results [55,100,101]. Fits with equivalent circuits to impedance spectroscopy data are ambiguous and physical interpretation can be arbitrary.

Extracting physically meaningful material parameters from these experimental techniques requires therefore numerical simulation. Numerical simulation provides a deeper understanding of the underlying physical processes.

Often JV-curves are fitted by simulation to extract charge transport parameters [22,29–32]. We showed in a previous publication that fitting JV-curves is clearly insufficient to unambiguously determine physical parameters [9]. Our conclusions are consistent with Set et al. demonstrating that parameter extraction from JV-curve fits are arbitrary [119]. The parameters are correlated – parameter 1 can have the same influence on the JV-curve as parameter 2. The influence of the different parameters on the result is highly entangled. Parameter correlation can be reduced by combining several experimental techniques [9]. The combination of a variety of experiments leads to a broader understanding, a higher accuracy and a quantitative description of a semiconductor device.

We perform measurements on an organic bulk-heterojunction solar cell comprising PCDTBT:PC_70_BM (weight ratio 1:4) as active material to demonstrate parameter extraction by numerical simulation. The device has the structure: ITO (130 nm)/MoO_3_ (10 nm)/PCDTBT:PC_70_BM (85 nm)/LiF/Al (100 nm) and has a power conversion efficiency of 3.3%. Device fabrication is described in Reference [120] and in the supplemental information. All measurements were performed on the very same solar cell, fully automated within 35 minutes such that unintentional degradation between different measurements or changes in ambient conditions can be minimized. The automated measurement without changing the contacting probes and measurement within a short period of time is important to obtain a fully consistent set of experimental data. We measured four nominally identical devices and found very good reproducibility. Here, we show measurement data of one device. An IV-curve was measured at the beginning and at the end of the procedure to confirm that no degradation occurred during the measurement. All measurements were performed using the all-in-one measurement system Paios [121]. For the illumination in all experiments a white LED (Cree XP-G) is used.

The simulation is described in the section ‘Simulation Model’ of this publication. All model equations are listed in the SI. We use a rather ‘simple’ model (discrete transport and trap energies) to keep the number of unknown parameters low. For the fitting the Levenberg-Marquardt [122,123] algorithm is applied (see SI for details).

We use the following procedure to obtain the simulation parameters:

(1) The relative dielectric constant *ε*_*r*_ and the series resistance *R*_*S*_ are extracted from the capacitance-frequency plot. The values are cross-checked with the displacement current in dark-CELIV.
(2) The parallel resistance *R*_*P*_ is determined from the reverse current of the dark JV-curve and can be cross-checked with the conductance of impedance spectroscopy data.
(3) The photon-to-charge conversion efficiency *η*_p2c_ is estimated from the short-circuit current.
(4) Electron and hole mobilities are fitted to the normalized transient photocurrent rise and decay.
(5) The injection barriers and the built-in voltage are fitted to the illuminated JV-curve and CV measurements.
(6) The recombination pre-factor is adjusted to the CELIV-peak current.
(7) Global fitting is performed for fine-tuning the parameter set. The parameters from steps 1–3 (*ε*_*r*_, *R*_*S*_, *R*_*P*_ and *η*_p2c_) were fixed during the global fitting routine.

**Figure 16.**
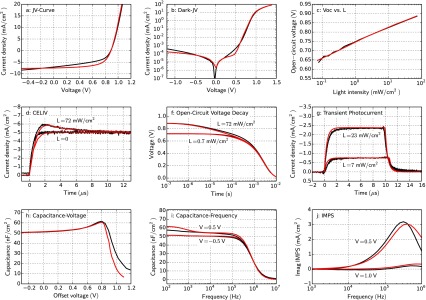
Measurements of an organic PCDTBT:PC_70_BM solar cell (black) and drift-diffusion simulation results (red) from a global fit. (a) JV-curve under illumination (*L* = 72 mW/cm^2^). (b) dark JV-curve. (c) Open-circuit voltage for varied light intensity. (d) Dark-CELIV (*L* = 0) and photo-CELIV (*L* = 72 mW/cm^2^) with ramp rate 100 V/ms. Light is turned off at *t* = 0. (f) Open-circuit voltage decay for two light intensities. Light is turned off at *t* = 0. (g) Transient photocurrent for two light intensities. Light is turned on at *t* = 0 and turned off at *t* = 10 μs. (h) Impedance spectroscopy at 10 kHz with varied offset-voltage. (i) Impedance spectroscopy at constant voltage with varied frequency. (j) Intensity-modulated photocurrent spectroscopy (IMPS) with constant offset voltage.

Figure 16 shows an overview of 9 experimental techniques with measurement and simulation. For all simulations the same material and device parameters are used, as summarized in Table 2. The simulation results (red curves) match the measurement data (black curves) very well. To the best of our knowledge, it is the first time that such a comprehensive description of an organic solar cell is published.

**Table 2.** Parameters that were used to simulate all experiments in Figure 16.

Parameter	Symbol	Value	Obtained by
Device thickness	*d*	85 nm	Measured by AFM
Device area	*S*	0.045 cm^2^	–
Series resistance	*R*_*S*_	90 Ω	High frequency range of capacitance-frequency plot
Parallel resistance	*R*_*P*_	160 MΩ	Reverse current of dark JV-curve
Relative permittivity	*ε*_*r*_	4.7	Capacitance level in capacitance-frequency plot and dark-CELIV
LUMO	*E*_LUMO_	3.8 eV	–
HOMO	*E*_HOMO_	5.37 eV	Fit
Band-gap energy	*E*_*g*_	1.57 eV	–
Workfunction MoO_3_	*Φ*_*A*_	5.22 eV	Fit
Workfunction Al	*Φ*_*C*_	3.88 eV	Fit
Built-in voltage	*V*_bi_	1.34 V	–
Effective density of states	*N*_0_	1.5⋅10^21^ cm^−3^	Fit
Electron mobility	*μ*_*e*_	1.6⋅10^−3^ cm^2^/Vs	Fit
Hole mobility	*μ*_*h*_	8⋅10^−4^ cm^2^/Vs	Fit
Langevin recombination efficiency	*η*	1.0	Fit
Photon to charge conversion efficiency	*η*_p2c_	0.37	Adjusted to match the short-circuit current
Electron trap density	*N*_*t*_	1⋅10^17^ cm^−3^	Fit
Electron trap depth	*E*_*t*_	0.4 eV	Fit
Electron trap – electron capture rate	*c*_*e*_	1⋅10^−11^ cm^3^/s	Fit
Electron trap – hole capture rate	*c*_*h*_	3.2⋅10^−10^ cm^3^/s	Fit

The illuminated JV-curve (Figure 16(a)) shows a slightly stronger voltage-dependence of the photocurrent than reproduced by simulation. This could be caused by field-assisted exciton dissociation (Onsager-Braun) [38,115] which was not included in the simulation but could be activated in the model for further refinement [22]. The dark JV-curve (Figure 16(b)) is well-described by the simulation. The open-circuit voltage dependence on light intensity (Figure 16(c)) shows an ideality factor of 1.2–1.5. Namkoong et al. [124] determined an ideality factor of 2 for a device with the same active layer. Such ideality factors can only be reproduced by introducing traps with SRH-recombination in the simulation model.

In the dark-CELIV (Figure 16(d)) no current-overshoot is observed indicating little or no doping. The current is mainly determined by RC-effects that are well-reproduced by the simulation. The photo-CELIV (Figure 16(d)) signal shows only a small overshot due to the high Langevin recombination in this system. The shape of the open-circuit voltage decay (OCVD), shown in Figure 16(f), is influenced by the amount of SRH-recombination and is reproduced well by the simulation for high (*L* = 72 mW/cm^2^) and low (*L* = 0.7 mW/cm^2^) light intensity (note the logarithmic time-scale). The voltage decay starting at 1 ms is caused by the measurement resistance of 1 MΩ which is also considered in the simulation. Figure 16(g) shows transient photocurrents for two different light intensities. The shape of current rise and decay is mainly influenced by the electron and hole mobility and are well reproduced by the simulation. The peak in capacitance-voltage (Figure 16(h)) is reproduced well by the simulation. There is, however, a small deviation in the injection regime (>0.8 V) that we cannot clearly attribute to a certain effect. Impedance spectroscopy data is shown in Figure 16(i) for two offset-voltages. The capacitance decay at high frequency (>300 kHz) is caused by the series resistance. The simulation reproduces the difference in capacitance for offset-voltages of −0.5 V and +0.5 V. The trapping leads to an increased capacitance at low frequency in the simulation that is slightly overestimated compared to the measurement. Discrete energy levels are used to describe the traps. A broader trap-distribution could reproduce the capacitance increase at low frequency more accurately [91]. Figure 16(j) shows intensity-modulated photocurrent spectroscopy (IMPS) data for two different offset-voltages. The IMPS data were not included in the global fit. The parameters determined from the global fit were used to simulate IMPS data as a cross-check. Indeed, the measurement and simulation of the IMPS signal fit reasonably well – a further indication for the validity of the approach for parameter extraction presented here. The correlation matrix of the global fit is shown in Figure S9 in the SI. Compared to the correlation matrix of a single JV-curve (Figure S10) the correlation is significantly reduced, indicating a high quality fit.

The parameters determined from the global fit are shown in Table 2 and allow conclusions about the material system under investigation. The system has high and balanced charge carrier mobilities leading to efficient transport. The mobilities observed here are higher than reported for similar material systems (5⋅10^−5^ cm^2^/Vs by CELIV and TOF [125] and 3⋅10^−4^ cm^2^/Vs by SCLC and DIT [113]). The reason might be the different morphology due to different processing. The Langevin pre-factor is 1, resulting in efficient recombination. It is consistent with the findings of Clarke et al. who determined a Langevin pre-factor between 0.3 and 1.0 for PCDTBT:PCBM which is common in polymer-fullerene material combinations [125]. An exception is P3HT:PCBM that shows a strongly reduced Langevin recombination with a pre-factor lower than 0.001 [13,114]. There seems to be no doping but a considerable density of electron traps leading to efficient recombination paths. Significant trap-assisted recombination has also been reported by Li and McNeill [126] and Clarke et al. [125] for PCDTBT:fullerene solar cells.

The photon-to-charge conversion efficiency is very low in this study. It can however also be caused by inaccuracies in the determination of the light intensity in our set-up. There is evidence for field-dependent exciton dissociation that lowers the photocurrent. The energy alignment of the contact materials to the HOMO and LUMO levels is very good leading to a high built-in voltage of 1.34 V and consequently to a high *V*_oc_.

The simulation and measurement results presented in this section show that material systems like PCDTBT:PC_70_BM can be described well even with a rather simple drift-diffusion model employing discrete transport and trap levels and Ohmic injection. All the main features observed in the experimental techniques can be reproduced. The simulation results provide physical insight and help to gain a better understanding of novel material systems and device concepts.

## 5. Summary

We present an overview of opto-electrical characterization techniques for solar cells, namely dark-CELIV, photo-CELIV, open-circuit voltage decay (OCVD), transient photovoltage (TPV), deep-level transient spectroscopy (DLTS), transient photocurrent (TPC), charge extraction (CE), impedance spectroscopy (IS), capacitance-voltage (CV), intensity-modulated photocurrent spectroscopy (IMPS), dark JV-curves and open-circuit voltage versus light intensity measurements.

Simulation results of all these techniques are presented on the basis of 10 common limitations and defects of solar cell devices. We provide rich information for judgement and interpretation of experimental results of these characterization techniques. Doping might be best extracted from dark-CELIV measurements. Recombination clearly influences the peak-height of the photo-CELIV current whereas the charge carrier mobility influences the rise-time in TPC. From the TPC decay and the DLTS decay, trap densities and trap depths may be estimated using temperature dependent measurements. The charge extraction experiment underestimates the effective charge carrier density by up to a factor of 5 in our simulations. The series-resistance and the electrical permittivity can be determined from capacitance-frequency plots of impedance spectroscopy data and from dark-CELIV. A capacitance-rise at low frequency is an indication of slow trapping. With capacitance-voltage measurements the injection behaviour can be studied. We recommend not to use Mott–Schottky analysis of CV data for thin devices like organic solar cells. The ideality factors from dark JV-curves and *V*_oc_ versus light intensity measurements are a clear indicator for trap-assisted recombination. Only the case with deep traps leads to an ideality factor of 2 in our simulations. The shunt resistance is extracted from the reverse current of the dark JV-curve or OCVD. The accuracy of the parameter extracted from these techniques using analytical approaches is discussed.

We further demonstrate comprehensive parameter extraction from experimental data by global parameter fitting on the example of an organic bulk-heterojunction solar cell comprising PCDTBT:PC_70_BM. Our simulation results match the data of 9 different experimental techniques in the steady-state, transient and frequency domain very well. Problematic parameter correlation is minimized by the combination of several techniques. All relevant parameters that govern charge transport are determined including the electron and hole mobilities, recombination pre-factor, trap density, trap depth, built-in potential, injection barriers, shunt resistance, series resistance and the relative dielectric constant.

We provide assistance in interpretation of experimental results and demonstrate comprehensive parameter extraction. Understanding and quantifying physical effects is a prerequisite for further progress in research of efficient and stable third-generation solar cell technologies.

## Disclosure statement

No potential conflict of interest was reported by the authors.

## Funding

This work was financially supported by COST Action MP1307 StableNextSol.

## Supplemental data

Supplemental data for this article is available online at https://doi.org/10.1080/14686996.2018.1442091.

## Supplementary Material

Supplementary.pdf
