# Supplementary material for: Opto-electronic characterization of third-generation solar cells
Source: Sci Technol Adv Mater. 2018 Mar 19;19(1):291–316. doi: 10.1080/14686996.2018.1442091 (PMC5917438; doi:10.1080/14686996.2018.1442091)
Supplement: Supplementary.pdf [file TSTA_A_1442091_SM5466.pdf]

Opto-electronic characterization of third generation solar cells Supplemental information

Martin Neukom, Simon Züfle, Sandra Jenatsch and Beat Ruhstaller

1. Measurement techniques

1.1. Transient photocurrent decay

In the manuscript we presented transient photocurrent simulations with rise and decay. Here we analyse the same TPC decay in detail. Figure S1 shows simulation results of the transient photocurrent decay for all cases defined in Table 1 in the manuscript. With a lower mobility (b) the decay is slower because it takes longer until the device is empty. In the case 'deep traps' (c) the current shows an undershoot (the photocurrent becomes positive). Charges flow back into the device. This reverse current can be explained by looking at the trap occupation. In the dark more traps are filled than under illumination. Illumination leads to a depletion of traps via SRH-recombination. The 'shallow traps' (c) lead to an exponentially decaying current caused by thermal emission of the carrier from the traps. Interestingly, the case with an extraction barrier (a) shows a very similar behaviour, as also here an energetic activation governs the slow part of the charge collection.

The tail current is integrated and divided through the device volume to obtain the charge carrier density as shown in Figure S1f. The grey lines represent the effective charge carrier density calculated from integrating the simulated charge carrier profiles at short-circuit. The charge carrier density at 0 Volt in the dark is subtracted. Similarly, as in the case of charge extraction also here the charge carrier density is underestimated by a factor of 10 or more due to charge carriers that recombine before they can be collected.

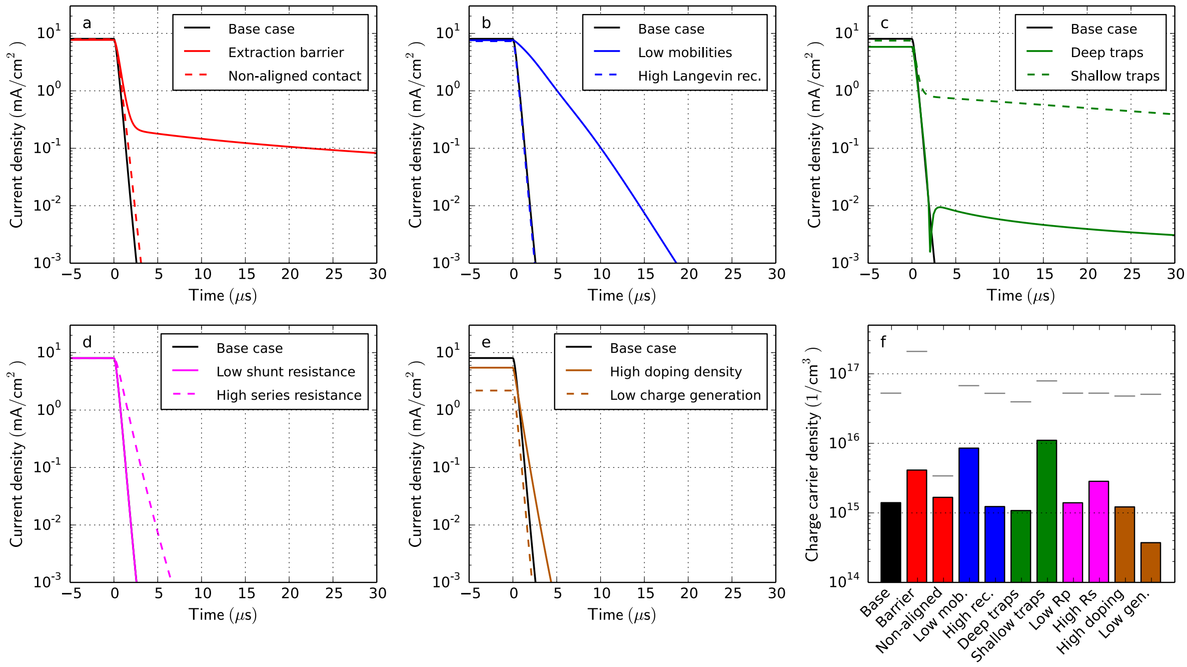

Figure S1: *Transient photocurrent decay simulations for all cases in Table 1. The light is turned off at t=0. f) Charge carrier density obtained from integration of the decay-current over time. The grey lines represent the effective charge carrier density from the simulated charge carrier profiles at short-circuit.*

1.2. Open-circuit voltage versus temperature

Measuring the open-circuit voltage V_{oc} down to low temperatures is an effective technique to estimate the built-in voltage [1]. Figure S2 shows simulations of V_{oc} versus temperature. Apart from the case 'extraction barrier' all curves reach exactly the built-in voltage at low temperature (< 50 K). At such low temperatures the open-circuit voltage is limited by the built-in voltage only. This method however only works as long as there is no extraction barrier. In the case 'extraction barrier' the V_{oc} collapses to zero because at low temperature the barrier cannot be overcome by charges.

If the contacts are perfectly aligned with the energy levels of the active layer, the V_{oc} would reach the band-gap energy at zero Kelvin. By linear extrapolation of the V_{oc} to 0 K the electrical band-gap can therefore be estimated. In Figure S2f we show the results of the extrapolation of the simulation data in the linear regime (250 K to 300 K). The effective band-gap of 1.57 eV (simulation input) is estimated accurately in all cases except for 'extraction barrier' and 'non-aligned contact'. In both cases the open-circuit voltage versus temperature is not yet in a linear regime at 300 K and higher temperatures would be required for the analysis.
We conclude that the band-gap estimation works precisely if the injection barriers are low.

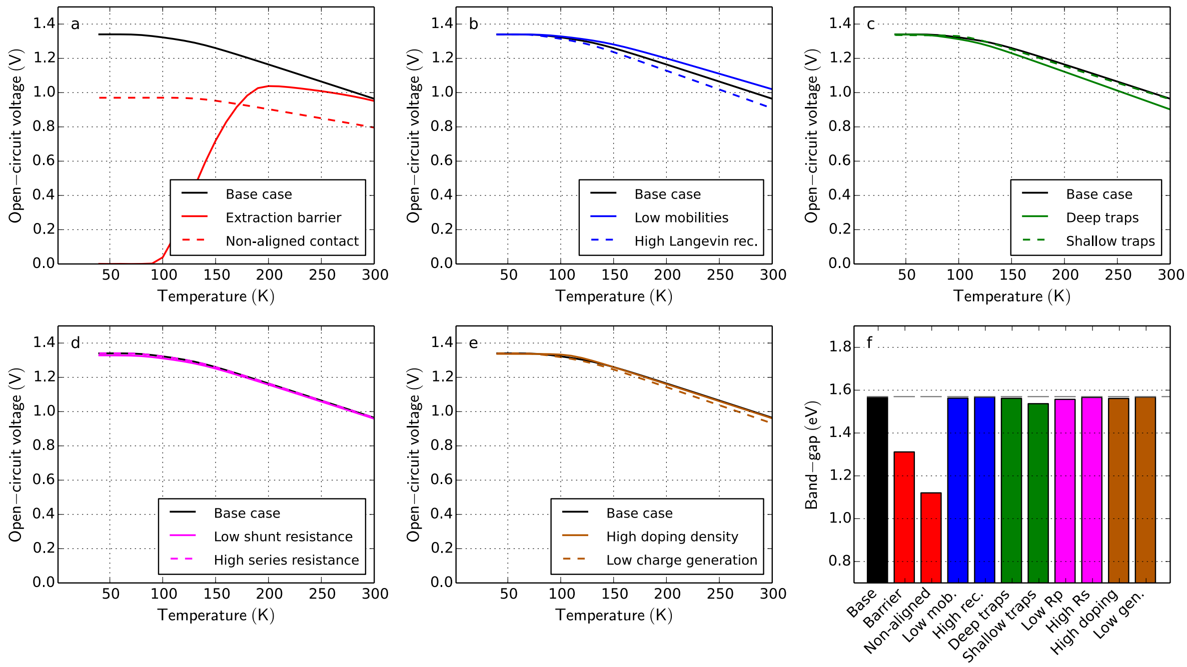

Figure S2: *Simulation of open-circuit voltage versus temperature for all cases defined in Table 1. Apart of the case 'extraction barrier' all cases reach exactly the built-in voltage at low temperature. f) Extrapolation of the linear regime at high temperature to 0 Kelvin. The effective band gap is 1.57 eV in all cases indicated by the grey lines.*

1.3. Intensity-modulated photovoltage spectroscopy (IMVS)

IMVS is a technique that is applied to study recombination. Compared to IMPS it is performed at open-circuit. In Figure S3 IMVS simulation results are shown for all cases. Figure S3f shows the charge carrier lifetime calculated from the frequency of the IMVS peak. The cases ‘extraction barrier’ and ‘non-aligned contact’ (a) show a very similar behaviour with a peak in the imaginary part. It might seem surprising that the case ‘high Langevin recombination’ (b) has a peak at the same frequency and consequently the same charge carrier lifetime. The reason is that the V_{oc} of the case ‘high Langevin recombination’ is lower at this light intensity. Plotting the charge carrier lifetime versus the V_{oc} is more conclusive, as we show in the section 1.4.

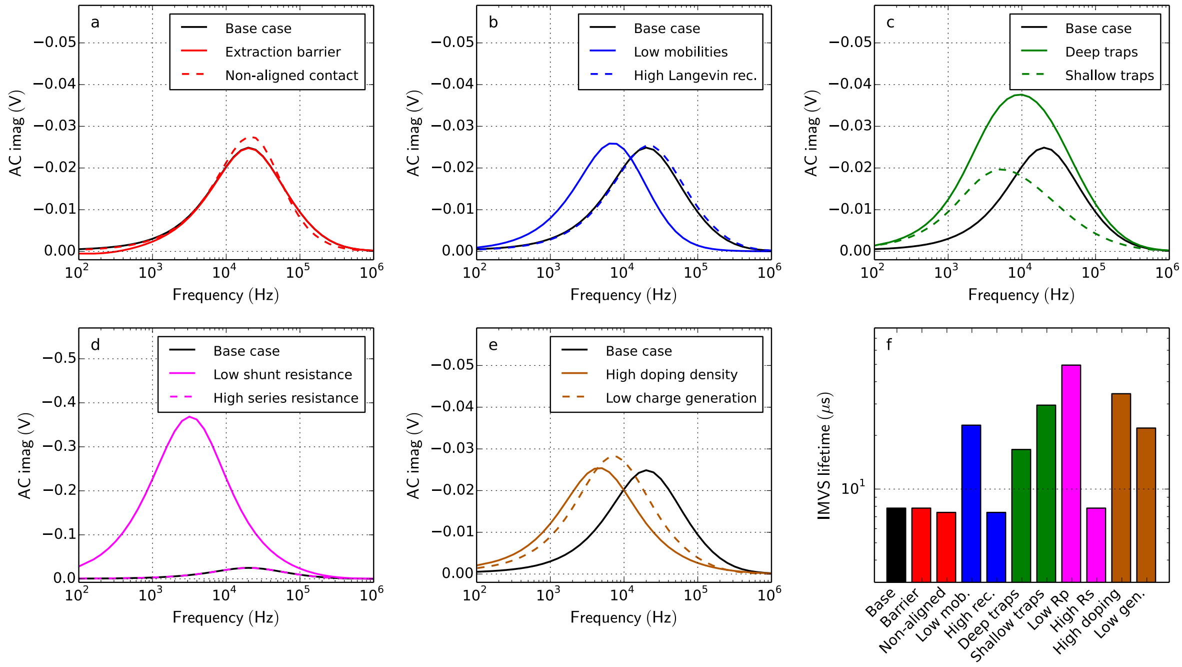

Figure S3: *Simulation of IMVS for all cases defined in Table 1. The offset light intensity is 3.6 mW/cm^2 and light modulation amplitude is 20% of the offset light intensity. f) IMVS charge carrier lifetime extracted from the peak frequency.*

1.4. Transient photovoltage

As we describe in the manuscript the quantity 'charge carrier lifetime' is not clearly defined in p-i-n structured solar cells. Nevertheless, we perform TPV simulations and extract charge carrier lifetimes from the exponential voltage decay after light turn-off. Figure S4 shows charge carrier lifetimes determined from TPV simulations versus the open-circuit voltage for varied offset light intensities. The lifetimes were corrected with the reaction order of 2.0 according to O'Regan *et al.* [2]. The points show the lifetimes extracted from IMVS simulations (see section 1.3) for two different offset light intensities. Also these lifetimes were corrected with the reaction order. Apart from minor numerical deviations in Figure S4c the lifetimes from TPV and IMVS agree completely. TPV and IMVS are therefore suited to cross-check measured charge carrier lifetimes. Another method to determine the charge carrier lifetime versus the open-circuit voltage is the calculation from the time-derivative of OCVD measurements (smoothing might be required) according to

$$\tau_{OCVD} = -\frac{n \cdot k_b \cdot T}{q} \cdot \left(\frac{dV_{oc}(t)}{dt}\right)^{-1},$$

where n is the ideality factor and $V_{oc}(t)$ is the transient OCVD voltage signal. In our simulations lifetime calculated from OCVD and from TPV agree approximately (not shown).

The ideal charge carrier lifetime τ under the assumption of bimolecular recombination and equal densities of electrons and holes can be described as

$$\tau = \frac{1}{\beta \cdot N_0} \cdot \exp\left(-\frac{V_{oc} \cdot q - E_g}{2 \cdot k_B \cdot T}\right)$$

where β is the recombination pre-factor, N_0 is the density of states, V_{oc} the open-circuit voltage, q the unit charge, E_g the band-gap, k_B the Boltzmann constant and T the temperature. The lifetime calculated from this equation is shown in grey using the recombination pre-factor of the 'base' case. In the 'base' case the charge carrier lifetime reaches exactly the analytical solution. The charge carrier densities are homogenous and therefore the lifetimes agree with the zero-dimensional analytical solution. At lower light intensity the charge carrier lifetime is underestimated by the simple formula. The reason is that the charge carriers are spatially inhomogeneous [3].

In the case 'non-aligned contact' (a) the V_{oc} is lower compared to the 'base' case but the charge carrier lifetime is the same. It does not agree with the analytical solution although it has only bimolecular recombination as in the 'base' case.

The case 'low mobilities' (b) agrees as well with its analytical solution at high light intensity. However, the case 'high Langevin recombination' does not reach the analytical solution. With higher recombination the inhomogeneity of the charge carrier distribution increases and the lifetime approach fails.

While the 'shallow traps' (c) lead to a higher V_{oc}, the 'deep traps' (c) lead to a reduced V_{oc} due to SRH-recombination. The shunt resistance (d) leads to a collapse of the V_{oc} but fairly similar lifetimes.

In Figure S5 we show the same lifetime data plotted versus the charge carrier density extracted from simulated charge extraction. Also here the analytical solution for the lifetime is drawn with a grey line. The assumption is an equal electron and hole density that is spatially homogenous with bimolecular recombination only. Also here it is shown that the simulated TPV lifetimes only agree with the analytical solution in the 'base' case at high light intensities. At low light intensities the charge carrier distribution becomes too inhomogeneous and the lifetime analysis fails. One might interpret such measurement results by a reduced recombination order at low light intensity – but in fact it is only a spatial separation that causes this effect. This conclusion agrees partly with Kiermasch *et al.* stating that apparent charge carrier lifetimes in thin solar cells are mainly caused by capacitive discharging [4].

We conclude that lifetimes plotted versus charge carrier density show the expected trends, but detailed conclusions about recombination and charge transport from such measurements are difficult.

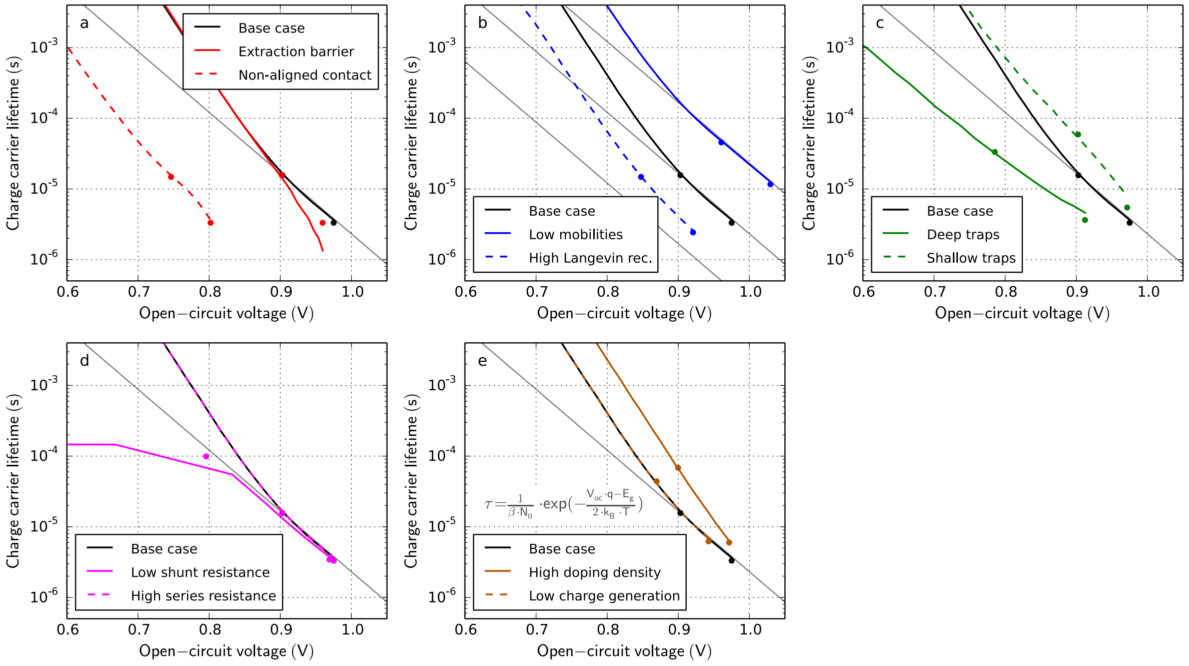

Figure S4: *Transient photovoltage simulations for all cases in Table 1. From an exponential decay fit to the simulation result the charge carrier lifetime is calculated. The points mark lifetimes calculated from IMVS at offset light intensities 65 mW/cm^2 and 3.6 mW/cm^2. All lifetimes are corrected with a reaction order of 2. The grey line shows the analytical solution for purely bimolecular recombination in a zero-dimensional model with equal electron and hole densities.*

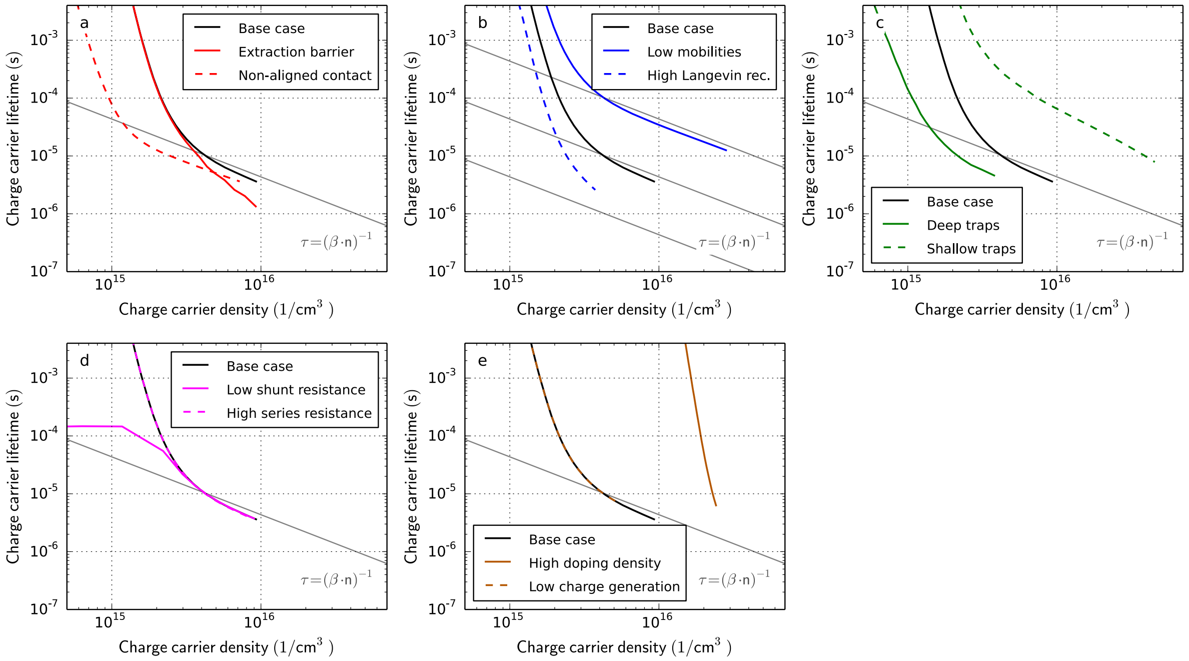

Figure S5: *Transient photovoltage simulations for all cases in Table 1 in combination with the charge carrier densities determined from charge extraction simulations. The grey line shows the analytical solution for purely bimolecular recombination in a zero-dimensional model with equal electron and hole densities.*

1.5. Impedance spectroscopy

In Figure S6 the same impedance spectroscopy data as in Figure 13 in the manuscript (capacitance-frequency) is shown in the Cole-Cole representation. In most cases one or more semicircles arise in this representation. Often the size of the semicircle is attributed to recombination in the device. The case 'high Langevin recombination' (b) shows however a larger semicircle than the base case. The size of the semicircle depends apparently on more factors than just the recombination. With traps, with the extraction barrier or with doping two semicircles show up in our simulations.

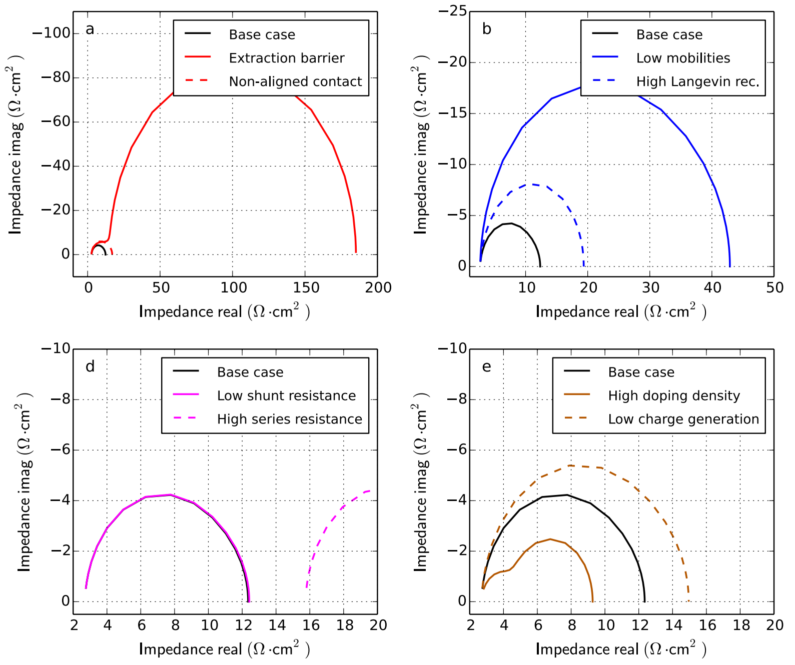

Figure S6: *Impedance spectroscopy simulation for all cases in Table 1 in Cole-Cole representation.*

1.6. Double injection transients

In double injection transients (DoI) the current response to a voltage step is measured. It is named double injection transients because electron and hole are injected. Figure S7 shows DoI simulations of all cases. Below 0.2 μs the current signal is dominated by the displacement current caused by RC-effects. In most cases it is followed by a slow current rise up to steady-state. The rise-time is related to charge transport and recombination. In the case 'low mobilities' the rise is therefore much slower compared to the 'base' case. Also the shallow and the deep traps lead to a slower current rise. In the cases 'high doping density', 'non-aligned contact' and 'high Langevin recombination' the current rise it too fast and hidden in the RC-effects.

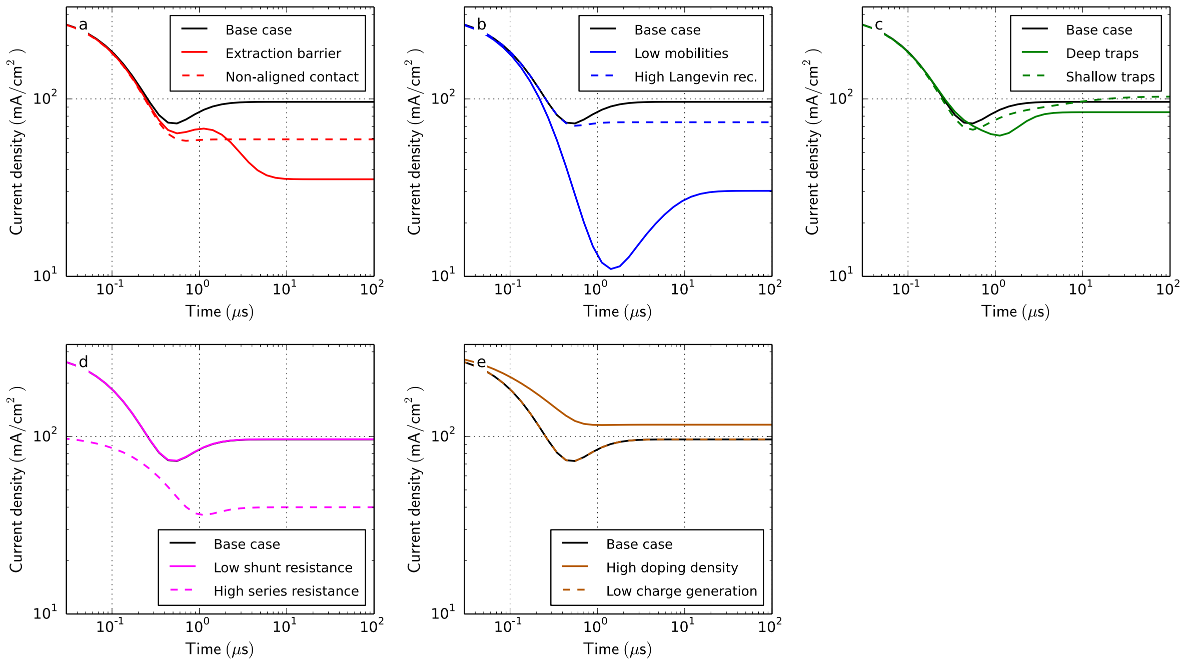

Figure S7: *Double injection transient simulations for all cases in Table 1. At t=0 the voltage steps from 0 V to 2 V. The simulation is performed in the dark.*

2. Imbalanced electron/hole mobilities

A common limitation in organic solar cells is an imbalance of the electron and hole mobilities. In such a case the slower carrier accumulates and leads to space-charge. In Figure S8 we show simulations of a solar cell with imbalanced mobilities and compare them with the ‘base’ case as described in the manuscript. In both cases the hole mobility is $4{\cdot}10^{-4}$ cm^2/Vs. The electron mobility is $2{\cdot}10^{-4}$ cm^2/Vs in the ‘base’ case and $2{\cdot}10^{-5}$ cm^2/Vs in the case ‘imbalanced mobilities’. In the case ‘imbalanced mobilities’ the electron mobility is lower by a factor of 20 compared to the hole mobility.

As seen in the JV-curve (Figure S8a) the fill factor is reduced in the case ‘imbalanced mobilities’. The slow electrons accumulate whereas the fast holes are quickly extracted. The resulting space-charge screens the electric field and hence the driving force for charge extraction. Therefore, the performance of the device decreases.

In Figure S8b the transient photocurrent response (TPC) is shown. The initial current rise and decay is governed by the fast carrier type (holes). In the case ‘imbalanced mobilities’ the current rise and decay shows a second, much slower component that is caused by the slower electrons. Two time-constants in TPC are an indication of imbalanced mobilities. A high charge carrier doping can however lead to a similar effect as shown in the manuscript.

Figure S8c shows photo-CELIV simulations of both cases. In the case ‘imbalanced mobilities’ the electrons are extracted later leading to a lower current peak and to a longer current tail. Whereas in the ‘base’ case most charges are extracted after 6 μs, the charge extraction lasts longer than 12 μs in the case ‘imbalanced mobilities’.

Figure S8d shows IMPS simulations of both cases. The different mobilities lead to two peaks with different time constants.

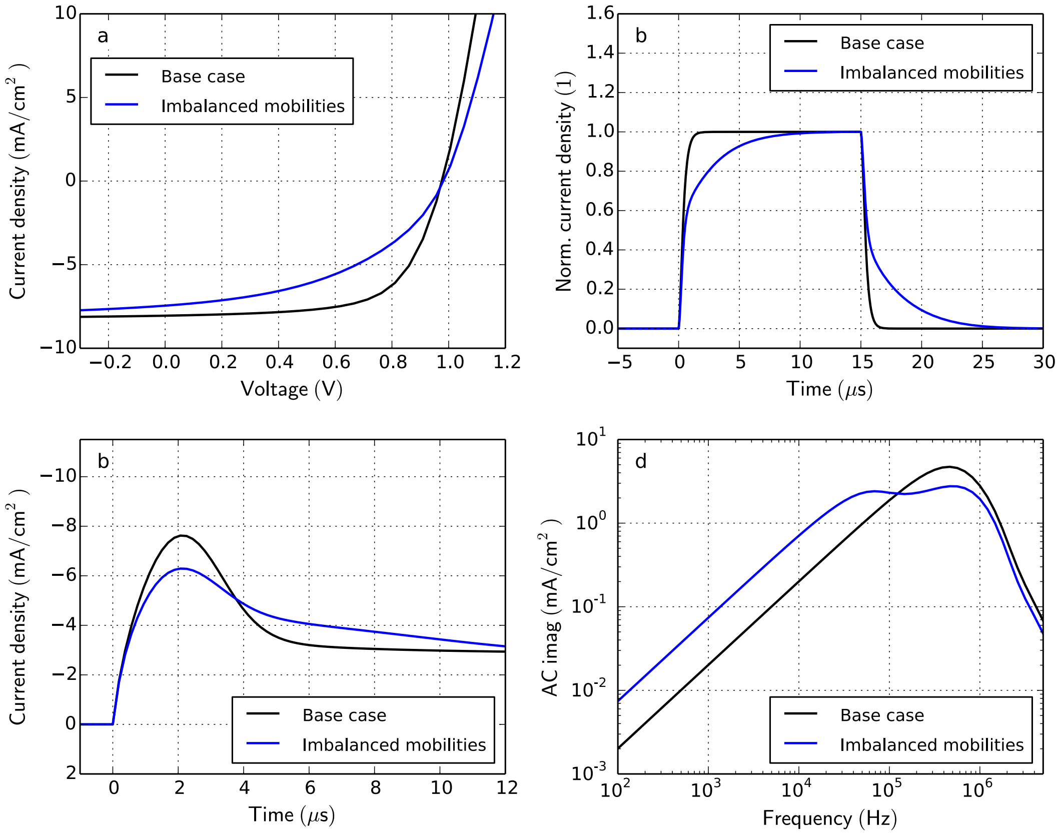

Figure S8: *Simulation results of the ‘base’ case and an additional case ‘imbalanced mobilities’. In both cases the hole mobility is $4{\cdot}10^{-4}$ cm^2/Vs. The electron mobility is $2{\cdot}10^{-4}$ cm^2/Vs in the ‘base’ case and $2{\cdot}10^{-5}$ cm^2/Vs in the case ‘imbalanced mobilities’. a) JV-curve simulations under illumination.*
b) Transient photocurrent simulations at 0 Volt. Light is turned on at t=0 and turned off at t=15 μs. The photocurrent is normalized to 1. c) Photo-CELIV simulations. The light is turned off at t=0 and the voltage ramp starts at t=0 with a ramp rate of 100 V/ms. The voltage offset prior to the ramp is set such that the current is zero at t<0. d) IMPS simulations. The offset light intensity is 3.6 mW/cm^2 and the light modulation amplitude is 20% of the offset light intensity.

3. Simulation

3.1. Simulation model

In this section the governing equations of the drift-diffusion model implemented in Setfos 4.5 [5] are explained. The quantities are described in the next section.

The continuity equation for electrons and holes governs the change in charge carrier density due to current flow, electron or hole exchange with traps, recombination and generation.

$$\frac{\partial n_e}{\partial t}(x,t) = \frac{1}{q} \cdot \frac{\partial j_e}{\partial x}(x,t) - R_{te}(x,t) - R(x,t) + G_{opt} \cdot g(x)$$

$$\frac{\partial n_h}{\partial t}(x,t) = -\frac{1}{q} \cdot \frac{\partial j_h}{\partial x}(x,t) - R_{th}(x,t) - R(x,t) + G_{opt} \cdot g(x)$$

For the calculation of the charge generation profile g(x) Setfos considers the measured illumination spectrum and the complex refractive indices and the thickness of each layer of the cell stack.

The Langevin recombination is proportional to the charge carrier densities of electrons and holes.

$$R(x,t) = \eta \cdot (\mu_e + \mu_h) \cdot \frac{q}{\varepsilon} \cdot n_e(x,t) \cdot n_h(x,t)$$

The currents of electrons and holes consist of drift in the electric field and diffusion due to the charge carrier density gradients.

$$j_e(x,t) = n_e(x,t) \cdot q \cdot \mu_e \cdot E(x,t) + \mu_e \cdot k_B \cdot T \cdot \frac{\partial n_e}{\partial x}(x,t)$$

$$j_h(x,t) = n_h(x,t) \cdot q \cdot \mu_h \cdot E(x,t) - \mu_h \cdot k_B \cdot T \cdot \frac{\partial n_h}{\partial x}(x,t)$$

The total current is the sum of electron current, hole current, displacement current and the current through the parallel resistance. This total current is constant in x.

$$j(x,t) = j_e(x,t) + j_h(x,t) + \frac{\partial E}{\partial t}(x,t) \cdot \varepsilon + \frac{V_{dev}(t)}{R_p}$$

The Poisson equation relates the electric field with the charges inside the layer.

$$\frac{\partial E}{\partial x}(x,t) = -\frac{q}{\varepsilon} \cdot \left(n_h(x,t) - n_e(x,t) - n_t(x,t) - n_{n-doping} + n_{p-doping}\right)$$

The integral of the electric field over x plus the built-in voltage is called device voltage. It is the applied voltage minus the voltage drop over the series resistance.

$$V_{dev}(t) = \int_0^d E(x,t) \cdot dx + V_{bi} = V_{applied}(t) - R_S \cdot j(t) \cdot S$$

The built-in voltage is defined as the difference in workfunctions of the electrodes. The workfunctions are calculated using the boundary charge carrier densities n_{p0} and n_{e0}.

$$V_{bi} = \frac{\phi_A - \phi_C}{q}$$

$$\phi_C = E_{LUMO} - \ln\left(\frac{n_{e0}}{N_0}\right) \cdot k_B \cdot T$$

$$\phi_A = E_{HOMO} + \ln\left(\frac{n_{p0}}{N_0}\right) \cdot k_B \cdot T$$

As boundary conditions the electron density at the anode and the hole density at the cathode are set to fixed values n_{e0} and n_{h0}.

$$n_e(0,t) = n_{e0}$$

$$n_h(d,t) = n_{p0}$$

The potential is evaluated according to

$$\varphi(x_1,t) = \int_0^{x_1} E(x,t) \cdot dx$$

Trapping and de-trapping of electron traps are described by the electron trap continuity equation. The electron trap can either exchange electrons with the LUMO level at the rate R_{te} or exchange holes with the HOMO level at the rate R_{th}.

$$\frac{\partial n_t}{\partial t} = R_{te} - R_{th}$$

Free electrons in the LUMO can be captured by traps. Trapped electrons can be released thermally activated into the LUMO.

$$R_{te} = c_e \cdot n_e \cdot (N_t - n_t) - c_e \cdot N_0 \cdot \exp\left(\frac{E_t - E_{LUMO}}{k_B \cdot T}\right) \cdot n_t$$

Trapped electrons can recombine with a free hole. An empty trap can capture an electron from the HOMO level by thermal activation (leaving behind a hole).

$$R_{th} = c_h \cdot n_h \cdot n_t - c_h \cdot N_0 \cdot \exp\left(-\frac{E_t - E_{HOMO}}{k_B \cdot T}\right) \cdot (N_t - n_t)$$

The three equations above describe SRH-recombination in a two-step process. Free electrons are captured in the trap and subsequently recombine with a free hole. Alternatively, an electron can be thermally activated from the HOMO to the trap level and from the trap level to the LUMO. The latter two routes occur however with lower probability.

3.2. Physical quantities

Quantity	Quantity	Unit
n_e	Electron density	cm^{-3}
n_h	Hole density	cm^{-3}
n_t	Trapped electron density	cm^{-3}
j_e	Electron current	mA/cm^2
j_h	Hole current	mA/cm^2
j	Total current	mA/cm^2
E	Electric field	V/m
φ	Potential	V
R	Recombination rate	$s^{-1}\ cm^{-3}$
R_{te}	Electron trap – electron exchange rate	$s^{-1}\ cm^{-3}$
R_{th}	Electron trap – hole exchange rate	$s^{-1}\ cm^{-3}$
g(x)	Charge generation profile	$s^{-1}\ cm^{-3}$
x	Dimension in layer direction	nm
t	Time	s
d	Layer thickness	nm
S	Device area	cm^2
μ_e	Electron mobility	cm^2/Vs
μ_h	Hole mobility	cm^2/Vs
η	Langevin recombination efficiency	1
V_{source}	Voltage of the voltage source that is connected to the device	V
V_{bi}	Built-in voltage	V
R_s	Series resistance	Ω
R_p	Parallel resistance	Ω
Φ_A	Workfunction of the anode	eV
Φ_C	Workfunction of the cathode	eV
E_{HOMO}	Energy of highest occupied molecular orbital	eV
E_{LUMO}	Energy of lowest unoccupied molecular orbital	eV
E_t	Trap energy	eV
n_{e0}	Electron density at the left electrode (x=0) as boundary condition of the simulation.	cm^{-3}
n_{p0}	Hole density at the right electrode (x=d) as boundary condition of the simulation.	cm^{-3}
N_0	Density of chargeable sites	cm^{-3}
N_t	Trap density	cm^{-3}
c_e	Capture rate for electrons	cm^3/s
c_h	Capture rate for holes	cm^3/s
G_{opt}	Photon-to-charge conversion efficiency<br>This factor accounts for non-dissociated excitons.	1
ε	Electrical permittivity ($\varepsilon = \varepsilon_0 \cdot \varepsilon_r$)	F/m
q	Unit charge	C
k_B	Boltzmann constant	J/K
T	Temperature	K

Table S1: *Physical quantities used in the equations of the previous section and in the main text.*

3.3. Simulation parameters

Table S2 summarizes all material and device parameters used to describe the cases defined in Table 1 in the manuscript. All cases are based on the case ‘base’. Only parameters that are different from the case ‘base’ are shown.

The case ‘extraction barrier’ comprises an additional layer between active layer and cathode. This additional layer has a LUMO level of 3.45 eV and a thickness of 3 nm. All other parameters of the extra layer are equal to the active layer.

**Parameter**	Base	Extraction barrier	Non-aligned contact	Low mobility	High rec.	Shallow traps	Deep traps	Low shunt resistance	High series resistance	High doping density	Low charge gen.
Device thickness (nm)	150										
Device area (cm^2)	0.045										
Series resistance (Ω)	60								350		
Parallel resistance (MΩ)	160							0.05			
Relative permittivity (1)	4.7										
LUMO (eV)	3.8										
HOMO (eV)	5.37										
Band-gap energy (eV)	1.57										
Workfunction Anode (eV)	5.22										
Workfunction Cathode (eV)	3.88		4.25								
Built-in voltage (V)	1.34		0.79								
Effective density of states (cm^{-3})	1.5e21										
Electron mobility (cm^2/Vs)	2e-4			2e-5							
Hole mobility (cm^2/Vs)	4e-4			4e-5							
Langevin recombination efficiency (1)	0.1				1						
Photon to charge conversion efficiency (1)	0.37										0.1
Doping density p-type ($1/cm^3$)	0									1e17	
Electron trap density (cm^{-3})	0					3e17	3e17				
Electron trap depth (eV)	-					0.3	0.8				
Electron trap – electron capture rate (cm^3/s)	-					3e-12	3e-12				
Electron trap – hole capture rate (cm^3/s)	-					3e-15	9.5e-17				

Table S2: *Simulation parameters of all cases defined in Table 1.*

4. Fitting procedure and parameter correlation

The parameter extraction in the manuscript is performed by fitting simulation results to the experimental data using the Levenberg-Marquardt [6,7] nonlinear least square algorithm. The Levenberg-Marquardt algorithm is a gradient method that minimizes a certain function.

4.1. Optimization

We define the functions f_i to be minimized as the difference between the simulated values s_i and the measured values m_i as

$$f_i = w_i \cdot (s_i - m_i),$$

where w_i are the weights for each point. The convergence of the fitting algorithm depends critically on the choice of measurement target m_i. We define several current-targets for different voltages on the JV-curve, current-targets at different times in transient experiments and amplitude and phase for each frequency point in impedance spectroscopy. Furthermore, we add some additional targets like the open-circuit voltage in the JV-curve and the time of the current-peak in photo-CELIV.
The weights w_i are chosen to set priorities for the fitting algorithm.

To calculate the next step in the optimization, the Jacobian matrix J of the function f_i is calculated according to

$$J_{ik} = \frac{\partial f_i}{\partial p_k},$$

where p are the simulation parameters to be optimized. The calculation of the Jacobian matrix is the computationally expensive step during the optimization.

The parameter-set h for the next step in the optimization is calculated by solving

$$(J^T \cdot J + \mu \cdot I) \cdot h = -J^T \cdot f,$$

where μ is a damping parameter and I is the identity matrix [6,7]. This procedure is repeated until the system has converged. Such a gradient method works well if the starting point is already close to the solution. The disadvantage of this method is that it might get stuck in a local minimum. Repeating the procedure from different starting points can be required to reach a good fit.

4.2. Parameter correlation

To judge the quality of the fit, we calculate a correlation matrix. The correlation matrix shows the linear correlation for all parameter combinations. It is normalized such that 1.0 means full positive correlation and -1.0 means full negative correlation. In case of full positive correlation increasing parameter 1 has the exact same influence as increasing parameter 2. The closer the value is to zero, the more independent are the parameters and the more unique the solution.

The correlation matrix M is calculated using the Jacobian matrix J from the section above according to

$$C = J^T \cdot J$$
$$M_{ik} = \frac{C_{ik}}{\sqrt{C_{ii} \cdot C_{kk}}},$$

where C is the covariance matrix. A more detailed explanation about the calculation of the correlation matrix can be found in our previous publication [8].

The correlation matrix of the global fit (Figure 16 in the manuscript) is shown in Figure S9. The diagonal of the correlation matrix is always one, since each parameter fully correlates with itself. Most parameters show only a very weak correlation with other parameters. The average correlation is 0.13. There are a few exceptions that show significant correlation. For example, the electron capture rate of the trap c_e correlates with the density of chargeable sites N_0. This can be explained looking at the governing equations in the previous chapter: Trap release to the LUMO-level is proportional to $c_e \cdot N_0$. Both parameters therefore have the same influence on the trap release current.

For comparison, we calculated the correlation matrix of only the illuminated JV-curve as shown in Figure S10. Here most parameters are highly correlated with other parameters and not a single parameter is independent. The average correlation is 0.50. Such a result indicates that the extracted parameters are not unique and a fit would therefore not be reliable. Comparing the two correlation matrices (Figure S9 and S10) it is clear that combining several experimental techniques reduces the correlation significantly.

Please note: The correlation matrix is calculated by linearizing a system at a particular working point. It is therefore only a local property of the system somewhere in the N-dimensional parameter space. At a different working point, the matrix might look different.
If there is strong correlation like in the case of Figure S10 the extracted parameters are most probably inaccurate. The opposite is however not true. A low parameter correlation as shown in Figure S9 is an indication for a good quality of the fit, but not a proof. It means that a stable local minimum has been found. No conclusions about a global minimum can be made.

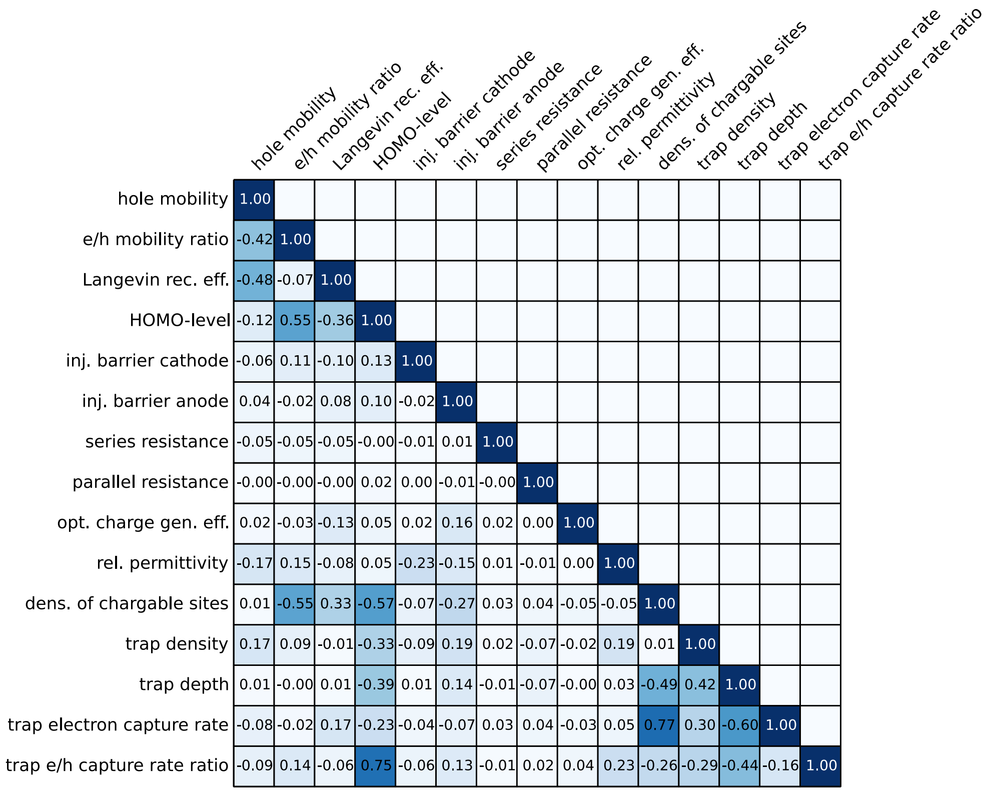

Figure S9: *Correlation matrix of the simulation results of Figure 16 in the manuscript. Experiments included in the calculation of the correlation matrix are: JV under illumination, dark-JV, V_{oc} versus light intensity, dark-CELIV, photo-CELIV, OCVD, TPC, CV and C-f.*

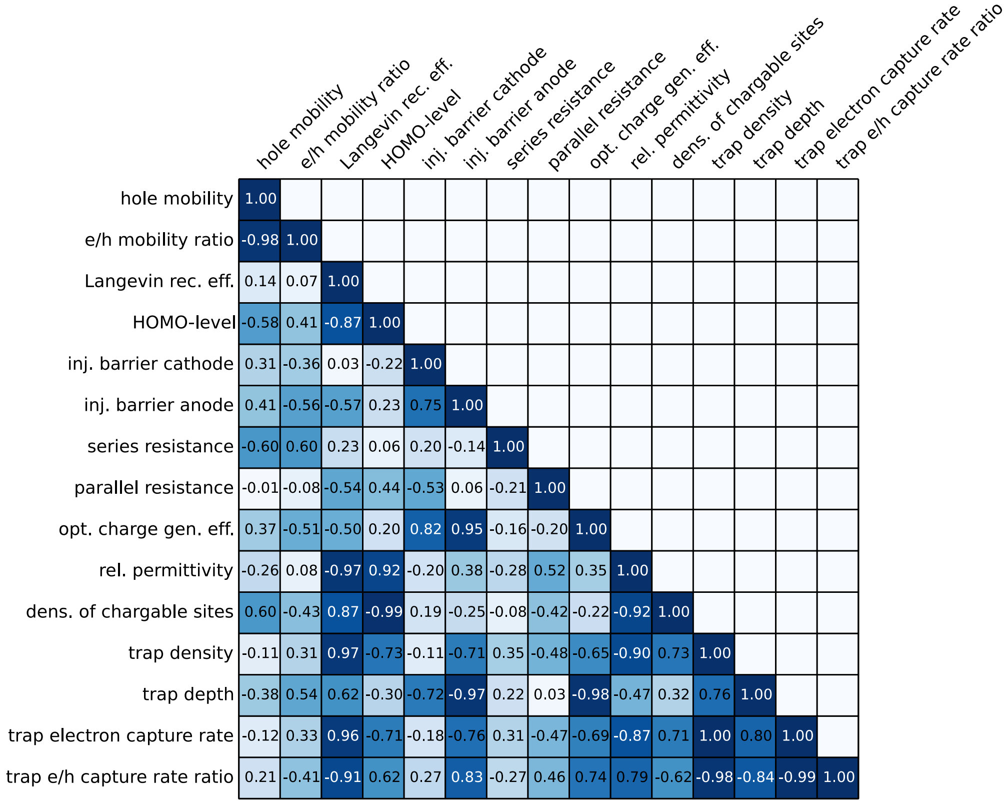

Figure S10: *Correlation matrix of the simulation results using only the illuminated JV-curve.*

5. Device fabrication

The device employed in the global fitting routine presented in the main text has the structure ITO (130 nm) / MoO_3 (10 nm) / PCDTBT:$PC_{70}BM$ (85 nm) / LiF / Al (100 nm) and was fabricated at Karlstad University. Below we give further information on the used materials and sample preparation.

5.1. Materials

PCDTBT (M_n = 19 kg/mol and M_w = 39 kg/mol) was purchased from Ossila Ltd, $PC_{70}BM$ (purity > 99%) was purchased from Solenne BV. MoO_3 (99.98%) was purchased from Sigma-Aldrich. Chlorobenzene (analytical grade) was purchased from Merck KGaA and used as received. Patterned ITO-coated glass substrates (100 nm, 20 Ω/sq) and light-curable encapsulation epoxy were purchased from Ossila Ltd.

5.2. Sample preparation

The solar cells were prepared on ITO-coated glass substrates that had been cleaned in isopropanol in an ultrasonic bath for 60 min and subsequently UV-ozone treated for 20 minutes. An 8 nm MoO_3 layer was deposited by thermal evaporation with a deposition rate of 0.4 Å/s at 10^{-6} mbar.
A blend solution of PCDTBT:$PC_{70}BM$ in a 1:4 weight/weight ratio, at a total concentration of 20 mg/ml, was prepared in chlorobenzene and filtered through a 0.45 µm polytetrafluoroethylene (PTFE) filter directly before being deposited on top of the MoO_3 by spin coating at 750 rpm for 100 s in a protected N_2 atmosphere (<0.1 ppm O_2, <0.1 ppm H_2O) inside a glove box (M. Braun Inertgas-Systeme GmbH). The active layer thickness was measured by scanning across a scratch in the film with the tip of an AFM (Nanoscope IIIa Multimode, Veeco Metrology group, now Bruker Corporation). After spincoating the active layer, the samples were transferred to the vacuum chamber of a thermal evaporator (Univex 350 G, Oerlikon Leybold Vacuum GmbH) integrated in the glove box, where 0.3 nm LiF and 100 nm Al was deposited with a deposition rate of 0.5 Å/s (LiF) and 1 Å/s (Al) at 10^{-6} mbar. Inside the glove box, the solar cells were encapsulated using encapsulation epoxy and glass coverslips. The epoxy was cured by exposure to UV-light (LV 202E, Mega Electronics) for 30 min [9].

6. References

[1] Mingebach M, Deibel C, Dyakonov V. Built-in potential and validity of the Mott-Schottky analysis in organic bulk heterojunction solar cells. Phys. Rev. B. 2011;84:153201.
[2] O'Regan BC, Durrant JR, Sommeling PM, et al. Influence of the $TiCl_4$ Treatment on Nanocrystalline TiO_2 Films in Dye-Sensitized Solar Cells. 2. Charge Density, Band Edge Shifts, and Quantification of Recombination Losses at Short Circuit. J. Phys. Chem. C. 2007;111:14001–14010.
[3] Deledalle F, Shakya Tuladhar P, Nelson J, et al. Understanding the Apparent Charge Density Dependence of Mobility and Lifetime in Organic Bulk Heterojunction Solar Cells. J. Phys. Chem. C. 2014;118:8837–8842.
[4] Kiermasch D, Baumann A, Fischer M, et al. Revisiting lifetimes from transient electrical characterization of thin film solar cells; a capacitive concern evaluated for silicon, organic and perovskite devices. Energy Environ. Sci. 2018;
[5] Semiconducting thin film optics simulator (SETFOS) by Fluxim AG, Switzerland [Internet]. Available from: http://www.fluxim.com.
[6] Levenberg K. A method for the solution of certain non-linear problems in least squares. Q. Appl. Math. 1944;2:164–168.
[7] Marquardt DW. An Algorithm for Least-Squares Estimation of Nonlinear Parameters. J. Soc. Ind. Appl. Math. 1963;11:431–441.
[8] Neukom MT, Züfle S, Ruhstaller B. Reliable extraction of organic solar cell parameters by combining steady-state and transient techniques. Org. Electron. 2012;13:2910–2916.
[9] Hansson R. Materials and Device Engineering for Efficient and Stable Polymer Solar Cells. Karlstads universitet; 2017.
